# scNPF: an integrative framework assisted by network propagation and network fusion for preprocessing of single-cell RNA-seq data

**DOI:** 10.1186/s12864-019-5747-5

**Published:** 2019-05-08

**Authors:** Wenbin Ye, Guoli Ji, Pengchao Ye, Yuqi Long, Xuesong Xiao, Shuchao Li, Yaru Su, Xiaohui Wu

**Affiliations:** 1 Department of Automation, Xiamen University, Xiamen, 361005 China; 2 Software Quality Testing Engineering Research Center, China Electronic Product Reliability and Environmental Testing Research Institute, Guangzhou, 510610 China; 3 College of Mathematics and Computer Science, Fuzhou University, Fuzhou, 350116 China; 4 Xiamen Research Institute of National Center of Healthcare Big Data, Xiamen, China; 5 Innovation Center for Cell Biology, Xiamen University, Xiamen, 361005 China

**Keywords:** Single cell RNA-sequencing, Dropout imputation, Similarity measurement, Cell type clustering, Network propagation

## Abstract

**Background:**

Single-cell RNA-sequencing (scRNA-seq) is fast becoming a powerful tool for profiling genome-scale transcriptomes of individual cells and capturing transcriptome-wide cell-to-cell variability. However, scRNA-seq technologies suffer from high levels of technical noise and variability, hindering reliable quantification of lowly and moderately expressed genes. Since most downstream analyses on scRNA-seq, such as cell type clustering and differential expression analysis, rely on the gene-cell expression matrix, preprocessing of scRNA-seq data is a critical preliminary step in the analysis of scRNA-seq data.

**Results:**

We presented scNPF, an integrative scRNA-seq preprocessing framework assisted by network propagation and network fusion, for recovering gene expression loss, correcting gene expression measurements, and learning similarities between cells. scNPF leverages the context-specific topology inherent in the given data and the priori knowledge derived from publicly available molecular gene-gene interaction networks to augment gene-gene relationships in a data driven manner. We have demonstrated the great potential of scNPF in scRNA-seq preprocessing for accurately recovering gene expression values and learning cell similarity networks. Comprehensive evaluation of scNPF across a wide spectrum of scRNA-seq data sets showed that scNPF achieved comparable or higher performance than the competing approaches according to various metrics of internal validation and clustering accuracy. We have made scNPF an easy-to-use R package, which can be used as a versatile preprocessing plug-in for most existing scRNA-seq analysis pipelines or tools.

**Conclusions:**

scNPF is a universal tool for preprocessing of scRNA-seq data, which jointly incorporates the global topology of priori interaction networks and the context-specific information encapsulated in the scRNA-seq data to capture both shared and complementary knowledge from diverse data sources. scNPF could be used to recover gene signatures and learn cell-to-cell similarities from emerging scRNA-seq data to facilitate downstream analyses such as dimension reduction, cell type clustering, and visualization.

**Electronic supplementary material:**

The online version of this article (10.1186/s12864-019-5747-5) contains supplementary material, which is available to authorized users.

## Background

Single-cell RNA-sequencing (scRNA-seq) is fast becoming an established and powerful tool for profiling genome-scale transcriptome of individual cells and capturing transcriptome-wide cell-to-cell variability [1]. With recent technological advances in cost and throughput, it is now possible to routinely generate a plethora of diverse scRNA-seq data sets that can be used to cluster cells [2, 3], determine cell types and states [4, 5], reconstruct developmental trajectories and cell lineage progression [6, 7], and identify key genes involved in the cell fate decision making [6]. scRNA-seq data potentially enables the profiling of diverse and heterogeneous systems [8, 9], however, scRNA-seq technologies suffer from high levels of technical and biological noise due to inefficient mRNA capture. A key challenge underlying the analysis of scRNA-seq data is the “dropout” phenomenon that a large fraction of genes, typically 85–95%, are with zero or low count due to intrinsic stochastic dynamics of gene expression and technical factors such as capture and sequencing efficiency [10, 11]. Such dropout confounds the reliable quantification of lowly or moderately expressed genes and obscures relationships between highly expressed genes, resulting in extremely sparse count data and hindering downstream analyses.

Since almost all downstream analyses on scRNA-seq, such as differential expression analysis, cell clustering, and lineage reconstruction, rely on the gene-cell expression matrix [12], the choice of preprocessing techniques is very critical in the analysis of scRNA-seq data. One routine step for preprocessing of scRNA-seq data is the correction of the expression measurements due to dropout events to mitigate the noise in scRNA-seq data. However, many studies on cell type identification, visualization, and lineage reconstruction do not explicitly model for dropout events but simply remove genes with low abundance and cells with low coverage prior to downstream analyses [13]. Despite of the simplicity and straightforwardness, it is not an ideal solution because that lowly expressed genes, such as transcription factors and cell surface markers, may be of great interest, and removing cells may propagate the biased sampling of the original cell population [14]. High variability and dropout events inherent in all current scRNA-seq platforms impede the interpretation of the data. Therefore, there is a growing need for developing new computationally efficient methods to overcome these hurdles.

Several computation approaches have been proposed for imputing missing values in scRNA-seq data. To our knowledge, MAGIC [14] is the first publicly available method for dropout imputation in scRNA-seq data. It is a Markov affinity-based graph method based on the idea of heat diffusion, which corrects signals of genes by sharing information across similar cells. scImpute [15] estimates dropout probability for each gene in each sample by a mixture model and divides genes into two sets according to their population-wide expression distributions. It then adopts a linear regression model to impute dropout events based on expression profiles of the most similar cells. scImpute is able to distinguish dropout zeros from real zeros. However, it assumes an overall dropout rate for each gene, while the dropout rate of a gene may be varied across cells due to factors such as RNA-seq protocols and cell types [10]. Both MAGIC and scImpute rely on pooling expression profiles of genes across similar cells. However, this strategy may cause over smoothing and thus tends to discard inherent cell-to-cell stochasticity that represents meaningful biological variation in gene expression [16]. SAVER [16] estimates the true expression levels of genes using a Bayesian approach that borrows information across genes in the same cell. One advantage of SAVER over MAGIC and scImpute is that it provides a measure of uncertainty for the recovered values. Both MAGIC and SAVER globally alter signals for all genes including those not affected by dropouts, which may introduce new biases into the data [15]. Several approaches try to reduce noise by clustering and combining cells. For example, drImpute [17] is an ensemble method based on consensus clustering, which performs clustering for multiple times and imputes zeroes by the average value of similar cells. However, these methods lose the advantage and resolution of single cells [14]. There are also some methods that attempted to impute missing values through gene-gene relationships [18] or by employing bulk RNA-seq data [19]. However, they focus only on imputing unobserved expression events while fail to correct lowly expressed genes whose signals are also unreliably measured. Imputation methods using bulk RNA-seq data fail to capture the cell-to-cell gene expression heterogeneity, which may lead to a high level of expression variation, even across cells of the same type [20, 21] or the same cell line [22, 23]. More importantly, most of these methods impute zeroes using measured information in the same data, which may amplify biases inherent in the data set [24]. Consequently, similar cells become more similar after imputation because of the increased similarities in imputed genes resulted from expression profiles of non-dropout genes.

Another promising approach to mitigate challenges of high variability and dropout events inherent in current scRNA-seq platforms is to analyze the scRNA-seq data in the context of molecular networks. In an effort to compile a comprehensive profile of biological modules underlying cellular composition and function, large interaction networks, such as protein-protein interaction (PPI) networks and metabolic pathways, continue to be systematically established for many model species [25, 26]. Proper integration of these networks with the scRNA-seq data and/or other high-throughput genomics data provides unprecedented resources for both biological and computational researchers to decipher the cell at a systems level. Topological structures of molecular networks have been exploited for function prediction according to the “guilt by association” principle, which assumes that genes that are colocalized or share similar topological characteristics in the interaction network tend to be functionally related [26, 27]. With the advance in high-throughput experimental techniques, large-scale interaction networks encapsulate rich sources of information, enabling approaches to infer functional patterns of unknown proteins by propagating knowledge from similar but better understood genes or proteins [26, 28, 29]. Recently, the topology of molecular networks was leveraged to infer gene expression measurements for scRNA-seq data. Ronen at el. [24] presented a network-diffusion based method called netSmooth for data denoising and imputation, using prior knowledge from PPI networks. This study demonstrated that the incorporation of meaningful information from massive experiments in the preprocessing of scRNA-seq data contributes to tempering experimental data with high noise and variability.

Despite of the great merit of molecular or functional interaction networks, their growing scale and complexity pose new challenges to biologists. For example, PPI networks tend to have a high false-positive rate and/or false-negative rate [30]. Second, PPI networks are typically sparse and have skewed degree distribution, which places a hurdle for algorithms dependent on neighbour information [31, 32] or those designed for networks with relatively uniform degree distributions [33]. Moreover, reference molecular networks from public databases are not data set- or sample- specific. Consequently, smoothing biological signals through gene interactions not present in the given sampled single cells can cause the aggregation of errors inherent in passenger measurements and the contamination of specific pathway signals, resulting in poor signal correction. Therefore, it is critical to distinguish whether gene interactions are functional in the investigated single cells or not. Fortunately, the ability to simultaneously profile thousands of genes and cells at single cell resolution provides the possibility to obtain gene-gene interactions from the scRNA-seq data, which enables learning context-specific interaction patterns that are only present in the given data set. Molecular networks from public domains encapsulate our knowledge of how genes and proteins interact in the cell universally, while the topological landscape in the context of the given scRNA-seq data expands our ability to explore the expression patterns in the relevant cells. Both data sources provide valuable information for inferring biological signals of genes or proteins. However, unique connectivity patterns of individual networks place new challenge on the systems level integration of heterogeneous sources of information to attain more precise inference. Given all that challenges, it is imperative to develop integrative approaches that can jointly combine priori interaction networks and the investigated sample-specific scRNA-seq data for scRNA-seq preprocessing to facilitate comprehensive downstream analyses, which can capture both shared and complementary information from diverse data sources.

We have presented scNPF, an integrative scRNA-seq preprocessing framework assisted by network propagation and network fusion. scNPF can be used as a general and flexible preprocessing step prior to downstream analyses of scRNA-seq data for recovering gene expression loss, correcting gene expression measurements, and learning similarities between cells. scNPF leverages the large sample size of scRNA-seq data to share information across similar cells, and in the mean time, jointly incorporates priori knowledge derived from molecular interaction networks, to impute gene expression for any given cell. Unique to our method is the ability to take advantage of not only the rich structures stored in biology networks but also the context-specific information from the investigated data to augment gene-gene relationships in a data driven manner. We demonstrated the ability of scNPF to amplify biological signals and derive similarity matrix across a wide spectrum of scRNA-seq data sets from various sequencing protocols. scNPF is a versatile preprocessing tool that can be used as a plug-in architecture for other standard tools for downstream analyses of scRNA-seq data.

## Results

### Overview of the integrative framework

**Fig. 1:**
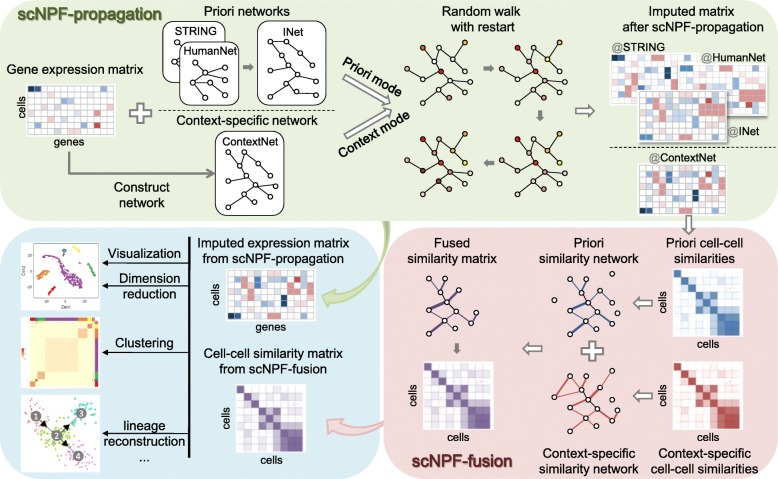
Schematic diagram of the scNPF framework. scNPF consists of two modules, scNPF-propagation for imputing dropouts `and scNPF-fusion for fusing multiple smoothed expression matrices to a cell-to-cell similarity matrix. Outputs from scNPF-propagation and scNPF-fusion can be used for downstream analyses of scRNA-seq data, such as visualization, dimension reduction, clustering, and lineage reconstruction

The basic scNPF framework consists of two modules (Fig. 1), including scNPF-propagation for imputing dropouts and scNPF-fusion for fusing multiple smoothed expression matrices to learn a cell-cell similarity matrix. The scNPF framework is highly integrative and flexible in that the two modules are independent but interconnected. scNPF-propagation involves a network propagation process based on random walk with restart (RWR) on a given gene-gene interaction network to obtain the distribution for each node (gene), which captures its relevance to all other genes in the network. This process takes the global connectivity patterns of the interaction network into account for profiling the topological context of each gene. More importantly, this module contains two modes of propagation, including the priori mode that uses a publicly available interaction network and the context mode that is solely based on the given scRNA-seq data set. The output of scNPF-propagation is a propagated gene-cell expression matrix, which could be used as input for scNPF-fusion. scNPF-fusion constructs a sample-similarity network for each propagated expression matrix and then integrates different networks into a single cell-cell similarity network based on a nonlinear combination method. The learned similarity matrix from scNPF-fusion or the smoothed expression matrix from scNPF-propagation can be used as inputs for other existing scRNA-seq pipelines or tools for downstream analyses, such as cell type clustering, dimension reduction, and visualization.

### Dropout imputation using scNPF

The scNPF-propagation module is capable of imputing missing expression values and smoothing non-zero expression measurements to recover the true signal for each gene in each cell. The output matrix of scNPF-propagation is of the same format as the original gene-cell expression matrix. Here we benchmarked scNPF-propagation on eight published scRNA-seq data sets (Additional file 2: Table S1) and compared scNPF-propagation with other popular imputation tools including MAGIC [14], scImpute [15], and SAVER [16]. Each method was applied to the raw expression matrix to obtain an imputed expression matrix. Here, the context mode of scNPF-propagation was used for imputation, which imputes dropouts and adjusts expression measurements solely based on the intrinsic structure of the given data without using any priori interaction network.

To examine the dropout phenomenon, we plot the expression levels of two randomly selected cells from the cortex fetal-quiescent cell type of the Darmanis data as an example (Additional file 1: Figure S1). Even though the two cells are from the same cell type, numerous genes are only detectable in one cell. This problem is mitigated by all imputation methods in that missing values of many genes were imputed and the Pearson correlation between the two cells increases (Additional file 1: Figure S1a). Surprisingly, MAGIC achieves an extremely high correlation (cor = 1) which may be due to additional spurious correlation introduced by this method. Previous studies [15, 16] pointed out that MAGIC may induce excess large counts that are absent in the raw data, leading to the loss of the biological variation between cells. Indeed, as can be seen from the t-SNE (T-distributed Stochastic Neighbour Embedding) [34] visualization (Additional file 1: Figure S1b) and the violin plots showing expression profiles of three marker genes among nine cell types (Additional file 1: Figure S2a), MAGIC tends to introduce artificial signals that alter the cell and the gene expression distributions greatly. Using scImpute, the Pearson correlation between the two cells increases greatly from 0.4 to 0.72 (Additional file 1: Figure S1a), while the t-SNE plot shows worse separation of cells compared to other methods or the raw data (Additional file 1: Figure S1b). Previously, a permutation study also revealed that the correlation estimates for gene pairs without biological correlation were potentially inflated by MAGIC and scImpute [16]. SAVER does not have a clear impact on the data, with a slight increase of correlation (from 0.4 to 0.42). In contrast, scNPF obtained higher correlation of the two cells than SAVER. Particularly, the 2D structure from SAVER is quite similar to that from scNPF (Additional file 1: Figure S1b), even though these two methods utilize completely different strategies for imputation. A comparison of expression profiles of three marker genes between the raw data and the imputed data by different methods also demonstrates that the imputation by scNPF and SAVER best reflects the gene expression signatures of the raw data (Additional file 1: Figure S2a). As expected, the number of expressed genes is greatly increased after imputation by all methods (Additional file 1: Figure S2b). However, again, the gene number distribution by MAGIC or scImpute is altered greatly compared to the raw data while scNPF and SAVER preserve the distribution. These results demonstrate that scNPF can recover the true gene expression signatures, and meanwhile, preserve the underlying data structures.

**Fig. 2:**
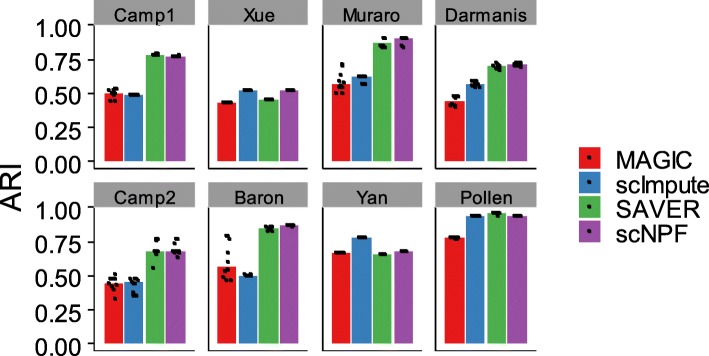
Benchmarking of scNPF-propagation on eight published scRNA-seq data sets.Clustering is performed by applying a consensus clustering method called SC3 on the imputed expression matrices. SC3 clustering is repeated for 10 times. Each dot represents an individual SC3 clustering run and each bar represents the median performance. ARI is employed to measure the concordance between inferred and true cluster labels. Detailed information of the data sets is shown in Additional file 2: Table S1

Next we assessed imputation results of scNPF-propagation by investigating the accuracy of single cell clustering. The cell clustering was carried out with SC3 [35], a popular clustering procedure that has been shown to provide most favourable results among various clustering methods. We assessed the clustering accuracy on the recovered data sets by four performance metrics including ARI (Adjusted Rand Index), Jaccard, Purity, and NMI (Normalized Mutual Information). All of these metrics range from 0 to 1, with a larger value indicating a higher match between the clustering result and the ground truth. t-SNE was adopted for visualization. According to the ARI index, for the eight data sets attempted, scNPF outperforms all other individual methods in five data sets (Xue, Muraro, Darmanis, Camp2, and Baron); scNPF performs better than at least two individual methods in all data sets except for the Pollen data (Fig. 2). For the three data sets (Camp1, Yan, and Pollen) where scNPF is not the best according to ARI, the ARI score of scNPF is always the close match of the best. For example, ARI scores from SAVER and scNPF on the Camp1 data are comparable (0.783 from SAVER, 0.772 from scNPF), which are much higher than that from MAGIC or scImpute. Similar results were obtained considering the other three metrics, including Jaccard, Purity, and NMI (Additional file 1: Figure S3). Generally, scNPF achieved higher or comparable performance than competing methods, whereas MAGIC and scImpute had a consistently lower performance. This result demonstrates that scNPF improves the cell type clustering by imputing dropout events in scRNA-seq data and also suggests the robustness of the performance of scNPF across various data sets.

scNPF-propagation has one adjustable parameter *rϵ*(0, 1), denoting a restart rate of the random walker (see Methods). *r* = 1 means no smoothing; a smaller *r* indicates higher level of smoothing, which allows diffusing further in the network. Previous studies have shown that the random walk process is not sensitive to the actual choice of *r* over a sizable range [24, 36, 37]. In this study, we set *r* at 0.5 for all experiments. Here we also examined the effect of *r* by performing scNPF-propagation on two data sets with moderate and large sample size. SC3 clustering results on the imputed matrices from scNPF-propagation demonstrated that the performance is stable for different values of *r* (Additional file 1: Figure S4).

### Dropout imputation using scNPF with different gene-gene interaction networks

Two modes are provided in scNPF-propagation for smoothing expression values and imputing zeroes in the sparse scRNA-seq data. In addition to the context mode used in the above experiment, the priori mode of scNPF is capable of imputing missing values using publicly available gene-gene interaction networks. Here three priori gene-gene interaction networks including String, HumanNet, and INet (see Methods) were utilized for scNPF-propagation, respectively. As INet is an integration of four different networks, it possesses a higher number of nodes than String and HumanNet, and accordingly, a much larger number of edges (gene-gene interactions) are present exclusively in INet (Additional file 1: Figure S5). Although most nodes (genes) (14,497) are common in all the three networks, only a small portion of edges (77,370) are shared by them. First we investigated the change of sparsity of each imputed expression matrix after network propagation with different modes and/or different gene-gene networks. Take as an example the Darmanis data. A total of 2560 genes were observed to be expressed in more than half cells in the raw data set (Additional file 1: Figure S6a). After imputation based on the context mode or the priori mode with different interaction networks, the number of moderately expressed genes increased greatly. As expected, the imputation result based on INet presents most expressed genes (15,398) as this network is much larger than other networks. In contrast, the imputed matrix based on the context mode contains the lowest number of expressed genes, which is due to that the context-specific network constructed from the Darmanis data is much smaller than other priori networks. Similar trend was observed for the sparsity of different imputed expression matrices (Additional file 1: Figure S6b), where the imputed matrix based on the context mode is the sparsest while the matrix based on INet is the densest. The results based on HumanNet and String present comparable number of expressed genes or sparsity.

**Fig. 3:**
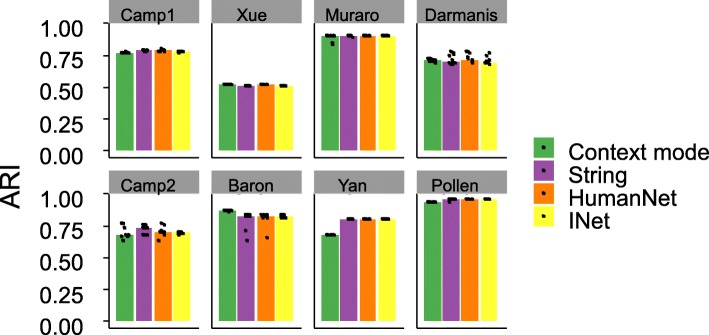
Benchmarking of scNPF-propagation on eight published scRNA-seq data sets using different propagation modes and/or priori networks. Clustering is performed by applying SC3 on the imputed expression matrices. SC3 clustering is repeated for 10 times. Each dot represents an individual SC3 clustering run and each bar represents the median performance. ARI is employed to measure the concordance between inferred and true cluster labels. Detailed information of the data sets is shown in Additional file 2: Table S1

Next we examined the impact of scNPF-propagation with different interaction networks on downstream SC3 clustering. First, we compared the performance using the context mode and the priori mode on the eight scRNA-seq data sets to examine the effect of propagation solely based on the given data set and propagation using publicly available interaction networks. Generally, scNPF yielded comparable results using different interaction networks across the majority of data sets (Fig. 3). Even that imputed matrices based on different interaction networks present highly variable sparsity (Additional file 1: Figure S6b, the Darmanis data), the cluster analysis showed comparable performance among these imputed matrices. This result indicates that although the context-specific interaction network constructed from the single cell data is much smaller than the priori networks obtained from public domains, imputation results from both kinds of networks are effective for downstream cluster analysis regardless of the scale of the network. Slight difference of the performance using different modes or different interaction networks was also observed. Specifically, for the Yan data, the performance of scNPF based on the context mode is lower than that using the priori mode (Fig. 3). This may be because that the relatively small sample size (90 cells) in the Yan data (Additional file 2: Table S1) makes the constructed context-specific gene-gene interaction network lack sufficient information to perform effective network propagation. In contrast, the performance for the Baron data which contains > 1000 cells using the context mode is higher than that using the priori mode (Fig. 3). Again, this result indicates that the context mode is potentially preferred for data with relatively large sample size, while the priori mode may be a better choice for data with small sample size. We note that even INet contains much more nodes and edges than other priori networks (Additional file 1: Figure S5), the performance based on INet shows no advantage over other networks. Particularly, for the Camp2 data, the ARI score based on the String network is slightly higher than that using INet or other networks (Fig. 3). It is probable that INet may contain more redundant gene-gene interactions than other networks, resulting in aggregation of errors during the network propagation. Despite of the slight difference among results using different networks, the performance of scNPF is quite stable in terms of various metrics (Fig. 3 and Additional file 1: Figure S7), demonstrating the robustness of scNPF using distinct propagation modes or different gene-gene interaction networks. As mentioned in the above benchmarking analysis that compares the context mode of scNPF with other imputation methods, the performance of scNPF is not the best according to ARI on three from the eight data sets (Camp1, Yan, and Pollen) (Fig. 2). By contrast, switching from the context mode to the priori mode with the String network, scNPF manifests the highest ARI score among all imputation tools on these three data sets (Camp1: SAVER = 0.783, scNPF-String = 0.787; Yan: scImpute = 0.774, scNPF-String = 0.803; Pollen: SAVER = 0.958, scNPF-String = 0.958) (Fig. 3). Taken together, scNPF is highly flexible in choosing different propagation modes and different interaction networks to adapt to diverse scRNA-seq data sets.

### Learning cell-cell similarities by scNPF

**Fig. 4:**
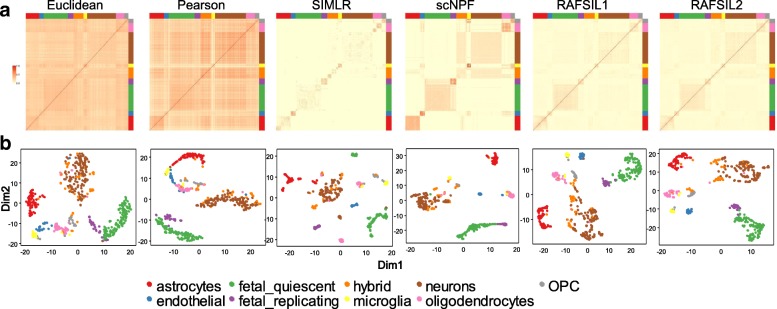
Benchmark results of scNPF-fusion on the Darmanis data. **a** Heatmaps for similarities learned from the data by Euclidean distances, pairwise Pearson correlations, SIMLR, scNPF-fusion, and RAFSIL. The scales in relative units denote the similarity. Cells with the same cell type (annotated by the colored axes) are grouped together. **b** t-SNE visualization for similarity matrices learned from different similarity measures. Each point denotes a cell. Smaller distance between two cells means higher similarity. True labels were not used as inputs for dimension reduction but were indicated in distinct colors to validate the results. RAFSIL1 and RAFSIL2 denote the result from the RAFSIL tool with the embedded RAFSIL1 or RAFSIL2 method

As a preprocessing tool, in addition to imputation, scNPF can also learn a cell-cell similarity matrix using the scNPF-fusion module. Free combinations of the raw expression matrices and/or the imputed expression matrices can be used as input for scNPF-fusion. We first compared the performance of the similarity metric learned from scNPF with other similarity measures including RAFSIL (a random forest based approach) [38], SIMLR (Single-cell Interpretation via Multikernel Learning) [39], Euclidian distance, and Pearson correlation by analyzing eight published scRNA-seq data sets. Here, scNPF takes the propagated matrices from scNPF-propagation using the context mode and the priori mode with the String network as inputs, and learns a matrix of similarities between cells by network fusion. Take the Darmanis data as an example. Apparently, the matrix with block structures learned from scNPF showed higher agreement with gold-standard labels than did other similarity measures (Fig. 4a). Block structures obtained by the correlation and Euclidian distance are indistinguishable from the background signatures; SIMLR generated far more blocks than the number of reference cell types; block structures learned from RAFSIL generally agree with the true structures except that the hybrid cells are indistinguishable. We also applied dimension reduction on scNPF’s similarity matrix to visualize differences between cell populations. The 2D embedding generated by scNPF is more consistent with true labels than other methods (Fig. 4b). Similar results were observed for the Baron data (Additional file 1: Figure S8) on which scNPF provides block structures with higher distinction than other distance metrics. Overall, scNPF generates more divergent clusters and individual clusters obtained by scNPF are more compact than other distance measures.

**Fig. 5:**
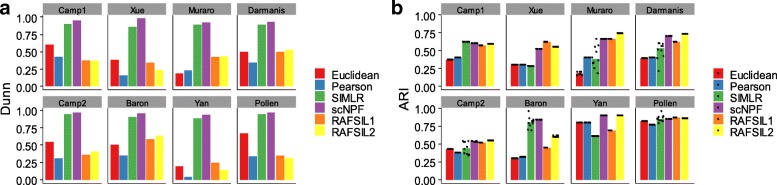
Performance comparison of the five similarity measurements on eight published scRNA-seq data sets. **a** The internal validation metric of Dunn was employed to measure the cell separation. **b** ARI is employed to measure the concordance between inferred and true cluster labels. K-means clustering is applied on the similarity matrices obtained from different methods. K-means clustering is repeated for 10 times. Each dot represents an individual K-means clustering run and each bar represents the median performance. Detailed information of the data sets is shown in Additional file 2: Table S1

In order to quantitatively measure the cell separation, three metrics independent of clustering methods, including Connectivity, DBI (Davies-Bouldin Index), and Dunn, were adopted to assess the cell separation (see Methods). Smaller values of DBI and Connectivity or larger values of Dunn mean better performance. According to the Dunn index, scNPF performs significantly better than other methods across all the eight data sets (Fig. 5a). For the Connectivity and DBI index, scNPF, SIMLR, and RAFSIL show comparable performance (Additional file 1: Figure S9). For the Connectivity index, scNPF outperforms all other individual similarity measures in four data sets (Additional file 1: Figure S9a). Under the DBI, scNPF provides the best result in three data sets and performs better than at least two methods in seven data sets (Additional file 1: Figure S9b). Even for those data sets where scNPF is not the best according to DBI, the performance of scNPF is always the close match of the best. For example, the DBI score from scNPF on the Camp1 data is 1.917, which is very close to the best score (1.902 from RAFSIL1). Generally, scNPF achieved higher or comparable performance than other methods, whereas Euclidean and Pearson correlation had a consistently lower performance. This result demonstrates the ability of scNPF-fusion in improving the cell separation and the robustness of scNPF across numerous data sets.

scNPF-fusion is a similarity framework which can also be flexibly adapted to any clustering methods that take similarities as inputs. Next we performed extensive comparisons of similarities learned from scNPF-fusion with other four similarity metrics by applying k-means for cell type clustering. According to the ARI score, clustering results demonstrate that similarities learned by scNPF and RAFSIL significantly outperform similarities obtained from Euclidean, Pearson correlation, and SIMLR (Fig. 5b). Specifically, scNPF provides the best or the second best ARI score for six from the eight data sets. Overall, scNPF shows similar performance with RAFSIL, while scNPF outperforms RAFSIL1 or RAFSIL2 in six from the eight data sets. Particularly, for the Baron data, scNPF presents much higher ARI score (0.835) than RAFSIL1 (0.446) or RAFSIL2 (0.608). In addition, we note that for several data sets (Muraro, Darmanis, Camp2, Baron, and Pollen), ARI scores of individual k-means clustering runs from SIMLR varied greatly, reflecting the poor robustness of SIMLR with k-means clustering. Similar results were observed using other three metrics including Jaccard, Purity, and NMI (Additional file 1: Figure S10). In addition to k-means, we also applied other clustering methods, including hierarchical clustering (HC) [40], spectral clustering [41], and partitioning around medoids (PAM) [42], to avoid potential bias of performance evaluation using different clustering methods. The performance of scNPF and RAFSIL is robust regardless of clustering methods used, whereas other three methods are more sensitive to clustering methods applied (Additional file 1: Figures S11-S13). For example, the performance of Euclidean and Pearson correlation using hierarchical clustering (Additional file 1: Figure S11) is much worse than that using other clustering methods (Additional file 1: Figures S10, S12, and S13). SIMLR with the spectral clustering (Additional file 1: Figure S12) presents much lower performance than with other clustering methods (Additional file 1: Figures S10, S11, and S13). In contrast, the performances of scNPF with different clustering methods are stable and consistently high across diverse data sets. These results demonstrate that the similarity matrix learned from scNPF is superior to and more robust than other similarity measures in clustering cell subpopulations.

The network fusion process in scNPF-fusion has three main parameters that could be tuned (see Methods): (i) *T*, the parameter controlling the number of iterations, usually between 10 and 20; (ii) *β*, a hyperparameter, usually between 0.3~0.8; and (iii) *K*, the number of neighbours, usually between 10 and 20. In this study, we set as default *K* = 20, *β* = 0.5, and *T* = 10 for all experiments. Similar to the parameter evaluation for scNPF-propagation (Additional file 1: Figure S4), here we examined the effect of these parameters by applying scNPF-fusion on the Darmanis and Baron data. Different combinations of these three parameters were tested. Results showed that scNPF-fusion is robust to a variety of parameter settings (Additional file 1: Figure S14).

### Learning cell-cell similarities by scNPF with different combinations of gene-gene networks

scNPF-fusion is able to fuse the propagated expression matrices obtained from different modes of scNPF-propagation, which allows to combine imputed data based on different interaction networks. Here we examined the impact of combinations of different networks on downstream clustering results. We defined six combinations of networks: combinations of any two priori networks from String, HumanNet, and INet; combinations of the network from the context mode and each of the individual priori networks. For each network combination, a similarity matrix is generated by scNPF-fusion. Take the Darmanis data as an example. Heatmaps show that block structures from similarities learned by scNPF-fusion with different network combinations are similar and in high agreement with gold-standard labels (Additional file 1: Figure S15a). The t-SNE visualization also demonstrates that similar 2D embeddings were generated with different network combinations and all cell types are clearly distinguishable (Additional file 1: Figure S15b).

**Fig. 6:**
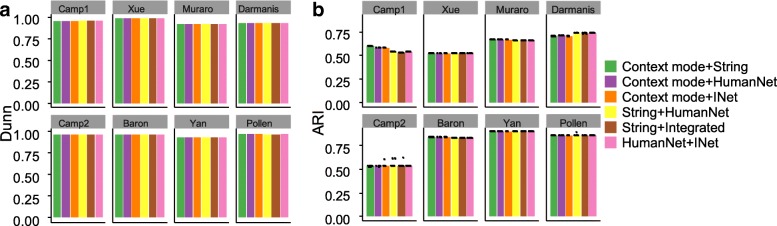
Performance comparison of similarities learned from scNPF-fusion with different network combinations on eight published scRNA-seq data sets. **a** Internal validation metric of Dunn was employed to measure the cell separation. **b** ARI is employed to measure the concordance between inferred and true cluster labels. K-means clustering is applied on the similarity matrices obtained from different methods. K-means clustering is repeated for 10 times. Each dot represents an individual K-means clustering run and each bar represents the median performance. Detailed information of the data sets is shown in Additional file 2: Table S1

According to the three internal validation metrics, the quantitative results measuring the cell separation on all eight data sets show little difference among these network combinations (Fig. 6a and Additional file 1: Figure S16). This result again demonstrates the robustness of scNPF based on different network combinations. Next we compared similarities learned from scNPF-fusion with different network combinations by applying k-means for cell type clustering. Generally, results are quite stable using different network combinations for most data sets (Fig. 6b). Slight difference was observed for the Camp1 data, where scores of ARI or other three metrics (Additional file 1: Figure S17) from network combinations including the context mode are slightly higher than scores from combinations without the context mode. This result indicates that even though results using different network combinations are comparable, incorporation of intrinsic structure learned from the given data (i.e., the context mode) and the structure from priori interaction networks (i.e., the priori mode) may generate better cell type clustering results than using solely priori interaction networks.

## Discussion

Due to the extreme sparsity and variability of scRNA-seq data, preprocessing of scRNA-seq data is a critical preliminary step prior to downstream analyses, such as dimension reduction, clustering, and spatio-temporal ordering of cells. The low read coverage, limited number of sampled cells combined with the technical biases and other data set specific variations in scRNA-seq data pose great challenges to single cell data analysis. Therefore, there is a growing need for developing reliable preprocessing methods to mitigate noise and dropouts in scRNA-seq data. Here we presented scNPF, a network-based integrative framework for preprocessing of scRNA-seq data by leveraging the context-specific topology inherent in the given data and the network information from priori gene-gene interaction networks. scNPF is a highly compact and flexible framework with two independent but connected modules, scNPF-propagation and scNPF-fusion. scNPF-propagation is able to construct a context-specific gene-gene interaction network from the given scRNA-seq data and perform network propagation by taking into account the global topology of molecular networks from public domains or the inherent structure of the investigated data. After propagation, dropout events in the sparse expression matrix are imputed and expression measurements especially lowly expressed ones are smoothed. Another module, scNPF-fusion, learns highly informative cell-cell similarities, considering diffusion patterns jointly for all smoothed networks from scNPF-propagation. Despite that output formats of these two modules are different (gene-cell expression matrix from scNPF-propagation; cell-cell similarity matrix from scNPF-fusion), they are both generic format that could be easily used by many other single cell tools. We have demonstrated the great potential of scNPF in scRNA-seq data preprocessing for accurately recovering gene expression values and learning cell similarity networks, which is critical for effective downstream analyses. We comprehensively evaluated the performance of scNPF using various scRNA-seq data sets spanning a variety of experimental technologies, cell types, tissues, etc. and compared it to other competing approaches, including MAGIC [14], scImpute [15], SAVER [16], RAFSIL [38], and SIMLR [39]. Results showed that scNPF achieved comparable or higher performance than competing approaches according to various metrics of internal validation or cluster accuracy. scNPF is powerful in mitigating noise caused by low efficiency, amplifying true biological relationships, and presenting better cell type identity.

The preprocessing approaches in the proposed scNPF framework have a number of desirable properties. First, by employing strategies of network propagation and network fusion, scNPF is able to take advantage of the fine-grained topology of ever-increasing molecular networks, which is particularly useful for well-annotated species with rich biological knowledge. Second, even for poor-annotated species with little knowledge of molecular network annotations, a context mode is provided in scNPF which constructs the sample-specific network solely based on the given data and leverages the learned network topology for correcting gene expression measurements. In this scenario, the propagation process is purely performed on the input scRNA-seq data, without any priori biological information involved. Third, scNPF-propagation implements an RWR-based universal framework that can freely take the gene-gene interaction network from different sources as the input. Particularly, the context-specific network construction in scNPF-propagation is independent of the RWR process, facilitating the incorporation of other newly developed tools for accurately inferring gene-gene networks. Fourth, scNPF is capable of combining context-specific gene-gene relations inferred from the given scRNA-seq data and interactions from priori molecular networks, which can yield better results by exploiting the advantages of the two distinct data sources (Fig. 6b and Additional file 1: Figure S16). Additionally, although scNPF-fusion is independent from scNPF-propagation, it can be performed on the layer of scNPF-propagation to learn similarity networks by jointly combining imputed and/or original gene expression matrices from different sources. As a universal preprocessing tool, scNPF can be seamlessly incorporated into most existing pipelines or standard tools for downstream analyses of scRNA-seq data, such as normalization [43], dimension reduction [44], clustering [45–47], differential expression analysis [10], and visualization [34]. Meanwhile, existing tools including those designed for dropout imputation [48–50] and those for reducing technical noise [11, 51] can also be easily incorporated into scNPF by applying the desired procedures before scNPF-propagation or scNPF-fusion.

Recent advances in high-throughput techniques have dramatically increased the availability of molecular interaction networks and stimulated the development of various algorithms to incorporate topological properties of these networks for elucidating cellular processes at the systems level. To the best of our knowledge, netSmooth [24] is the first method to impute dropouts by using priori molecular networks. However, it heavily relies on priori biological networks from public domains, which has several inherent limitations. One main challenge is the incomplete, sparse, and noisy nature of interaction networks [31, 32], consequently, the method is hindered by missing interactions and false positives in the networks. Second, related genes with little annotation are likely absent in the reference molecular networks obtained from public domains [37]. Moreover, these priori networks are not sample-specific, consequently, context-specific gene-gene interactions that are only present in the investigated data sets may be obscured by edges from the priori networks. Additionally, propagating information through gene interactions not present in the investigated cells may cause erroneous aggregation of passenger signals, leading to poor cell type stratification. Therefore, in addition to using priori molecular networks, it is important to take into account other relevant information, such as gene associations and expression patterns in the relevant cells. Fortunately, the increasing compendium of scRNA-seq data at unprecedented throughput and resolution enables us to extract context-specific patterns from functionally well-annotated genes to accurately recover gene expression signals of lesser-known ones. The priori biological knowledge from PPI networks in public domains and the context-specific information of gene-gene interactions from the given data set compensate each other, each of which encapsulates complementary information about associations missing in the other data source. Similar to many other network approaches [26], network propagation is the core component in scNPF for smoothing and amplifying signals in the same sub-network regions. Unlike the previous propagation-based approach (netSmooth [24]) which is solely based on priori known PPI networks, scNPF contains a context mode which models the information flow in the given cells to guide the direction of propagation in a context-specific manner. Although the gene-gene network from the context mode is much smaller than that from the priory mode (Additional file 1: Figure S6), results of scNPF-propagation based on both modes are comparable (Fig. 3 and Additional file 1: Figure S7). This may be due to that scNPF-propagation is performed in the context of a network, thus even two paths of information flow from different networks may have few genes or interactions in common, they could lead to similar cumulative effect on the same target gene. More importantly, scNPF enables propagating information along both the priori network in public domains and the context-specific network learned from the given data, which can integrate available data sources together from a gene network perspective to aggregate and amplify gene expression signatures in the highly sparse scRNA-seq data. Comprehensive results have demonstrated that this new strategy used by scNPF could improve performance of downstream analyses of scRNA-seq data, such as dimension reduction and cell type clustering (Fig. 2, Additional file 1: Figures S1 and S3).

The topological landscape of interaction networks from public domains or that learned from the give scRNA-seq data set provides a rich source of information for preprocessing of scRNA-seq data, however, an open challenge is how best to integrate multiple heterogeneous networks with different connectivity patterns. Most previous work focused on combining a collection of networks into a single network, which is normally achieved by summarizing the edges across different networks via algorithms like Bayesian inference [52–54]. While such methods are promising, a potential limitation is the substantial information loss caused by projecting diverse data sets onto a single network representation. Consequently, for instance, context-specific interaction patterns that are exclusively present in the network constructed from the given data set may be obscured by edges from other public data sources in the integrated network. To address this challenge, we proposed scNPF-fusion for scalable and robust network integration, which can be employed for fusing propagated results from individual networks. scNPF-fusion constructs similarity networks of cells for each (raw or propagated) gene expression matrix and then efficiently fuses them into a single similarity network, which takes advantage of the complementarities in diverse data sources to fully profile the spectrum of the underlying data. This integrated framework for joint learning heterogeneous networks can properly take advantage of the complete set of all interactions and associations from the priori network as well as the context-specific network learned from the given data, thereby increasing robustness to noise and enhancing accuracy for downstream analyses (Figs. 4, 5 and Additional file 1: Figures S7-S13). With the increased quantity and quality of the network data and scRNA-seq data, analysis of scRNA-seq data from a network perspective would provide deeper insight into the systems-level understanding of cellular processes at the single cell level.

## Conclusions

scNPF is a universal tool for preprocessing of scRNA-seq data, which jointly incorporates the global topology of priori interaction networks and the context-specific information encapsulated in the scRNA-seq data to capture both shared and complementary knowledge from diverse data sources. We have demonstrated the great potential of scNPF in scRNA-seq preprocessing for accurately recovering gene expression values and learning cell similarity networks across various scRNA-seq data sets. As an easy-to-use R package, scNPF can be used as a versatile preprocessing plug-in for most existing scRNA-seq analysis pipelines or tools to facilitate downstream analyses such as dimension reduction, cell type clustering, and visualization.

## Methods

### scRNA-seq data sets

We benchmarked scNPF-propagation on eight publicly available scRNA-seq data sets from human (Additional file 2: Table S1), reflecting a wide spectrum of sequencing protocols, sequencing depth, tissue origin, cell types, cell number, and heterogeneity of single cells. Except for the Xue data which was obtained from GEO (Gene Expression Omnibus) [55], other data sets in *SingleCellExperiment* format were downloaded from a repository of processed scRNA-seq data (https://hemberg-lab.github.io/scRNA.seq.datasets/). To reduce the computing time, we performed a conservative quality control (QC) process to remove low-abundance genes as suggested in other studies [14–16]. Genes whose mean expression level is smaller than a given cutoff (0.001 for the Muraro and Baron data sets; 0.01 for other data sets) or genes that are expressed in less than three cells were discarded.

### scNPF-propagation for imputing dropouts and correcting expression measurements

We adopted the network propagation approach based on RWR to smooth expression values, which takes into account both local and global topology of a given interaction network. Given a gene-gene interaction network *G* =  < *V*, *E*, *B*> with *V* as the set of genes and *E* as the set of interactions. Each entry *B*_*ij*_ in the transition probability matrix *B* stores the probability of a transition from node *i* to node *j*. The starting point is a vector *P*_0_ of scores (amount of information) on genes representing the gene expression profile of a given cell. After projecting the expression profile of a cell onto a molecular network, network propagation is applied to smooth the expression measurement across the network. Network propagation uses a process that simulates a random walk with restart, which can be computed iteratively according to the following function:

1 $$ {P}_{t+1}={rP}_0+\left(1-r\right){P}_tW $$

*P*_0_ is a restart vector recording the initial expression levels of genes in a given cell. *W* is a degree-normalized adjacency matrix of the gene interaction network. *W* is constructed by the adjacency matrix *B* and a diagonal degree matrix *D*, which is defined as *W* = *BD*^−1^. *r* is the trade-off between prior information and network diffusion, governing the distance that a signal is allowed to diffuse through the network during smoothing. The specific value of *r* has little effect on the results of network propagation over a sizable range. In this study, *r* is set at 0.5 for all experiments.

At each time point *t*, the random walk either flows from the current gene *u* to a randomly chosen neighbour *v* ∈ *V* or restarts at one gene in *P*_0_. The amount of information at each node *v* ∈ *V* depends on the sum of the information at the adjacent nodes of *v* at time *t* − 1, in proportion to the weights on the corresponding edges. When *t* is small, *P*_*t* + 1_ is close to the initial distribution *P*_0_. With the increase of *t*, the information propagates away from the prior distribution and reflects the network topology. The propagation function is run iteratively with sufficient steps until *P*_*t* + 1_ converges to a steady-state *P*:

2 $$ P=r{\left(I-\left(1-r\right)W\right)}^{-1}{P}_0 $$

After propagation, a smoothed expression profile is obtained for the given cell. The network propagation process is repeated for each cell in the gene-cell matrix to generate a new propagated matrix which is a much denser matrix with smoothed gene expression values.

Two modes of network propagation based on RWR, including the priori mode and the context mode, were proposed for imputing dropouts and smoothing expression measurements. In the priori mode, publicly available molecular networks are used for network propagation. In this study, three human gene interaction networks were obtained from different databases. The gene association network String (Search Tool for the Retrieval of Interacting Genes/Proteins) database (v9.1) [54] integrates protein-protein interactions from diverse sources, including computationally predicted interactions, physical and functional interactions. In the network, the weight of each link represents a combined score indicating the probabilistic confidence of associations between the proteins. The top 10% of edges of interactions ranked by the score were retained and genes whose summarized score of neighbours is 0 were removed. HumanNet (v1) [52] is a probabilistic functional gene network inferred from omics data collected in humans, yeast, worms, and flies, which adopts a Bayesian model to integrate different types of evidence into a single interaction score. INet [56] is an integrated network constructed from four existing human weighted gene interaction networks including String and HumanNet, which employs information entropy to define the uncertain degree of gene-gene links. For each network, the RWR-based network propagation was performed to propagate the scRNA-seq gene expression matrix through the network to obtained a new propagated matrix.

In the context mode, the network propagation is performed without any priori interaction network but solely relies on the given scRNA-seq data set. To this end, a context-specific gene-gene network is constructed from the scRNA-seq data set using the WGCNA package [57], a popular tool for constructing the weighted correlation network. First the count data was log2 transformed (pseudo count = 1). Then low-abundance genes that are below the 60th percentile of summarized expression levels in all cells were filtered out. Except for the Xue data which only contains 29 cells, low-abundance cells that are below the 50th percentile of summarized expression levels in all genes were discarded. Next, cells and genes with too many missing entries and genes with zero variance were further removed by the *goodSamplesGenes* function in the WGCNA package. Finally, the topological overlap matrix from the given expression data was obtained by the *TOMsimilarityFromExp* function in the WGCNA package, which was then used as input for igraph to construct a context-specific weighted and undirected network. Based on this context-specific network, the network propagation based on RWR can be performed to smooth the scRNA-seq gene expression matrix.

### scNPF-fusion to learn similarities by fusing multiple expression networks

We applied similarity network fusion (SNF) [58] to flexibly integrate two gene-cell expression matrices by constructing a network of cells for each input matrix and then fusing both networks into one comprehensive network. This process consists of two main steps for data integration. Here, we take the matrix from the priori mode of scNPF-propagation (hereinafter referred as *priorMatrix*) and the one from context mode (hereinafter referred as *contextMatrix*) as inputs for scNPF-fusion to demonstrate the process of learning a similarity matrix. First, scNPF-fusion constructs a cell-to-cell similarity matrix for *priorMatrix* and *contextMatrix*, respectively. Then, both similarity matrices are iteratively and gradually fused to a coherent and combined network, employing the non-linear method of message passing theory [59]. Finally, weak similarities which may be potential noise are discarded, and strong similarities are added. By generating consensus similarities among cells from *priorMatrix* and *contextMatrix*, SNF provides deeper insight into the comprehensive biological relationship among cells, beyond the scope of basic classification or subtyping methods.

Given a propagated gene expression matrix with *n* cells and *m* genes. A cell similarity network is denoted as a graph *G* =  < *V*, *E*>, where vertices *V* {*c*_1_,  … , *c*_*n*_} correspond to cells and edges *E* are weighted by how similar the cells are. Edge weights are described by a similarity matrix *W*_[*n* × *n*]_ with *W*_*ij*_ indicating the similarity between cells *c*_*i*_ and *c*_*j*_. The weight of an edge can be determined using a scaled exponential similarity kernel:

3 $$ {W}_{ij}=\exp \left(-\frac{d_{ij}^2}{{\beta \alpha}_{ij}}\right) $$

Here *d*_*ij*_ represents the Euclidean distance between cells *c*_*i*_ and *c*_*j*_. *β* is an empirical hyperparameter which is recommended to be set over a sizable range of [0.3, 0.8] [58]. *α*_*ij*_ is introduced to eliminate the scaling problem, which can be defined as follows:

4 $$ {\alpha}_{ij}=\left(\overline{d_{iN_i}}+\overline{d_{jN_j}}+{d}_{ij}\right)/3 $$

where *N*_*i*_ are the neighbours of cell *c*_*i*_ and $\overline{d_{iN_i}}$ is the mean distances between the cell *c*_*i*_ and each of its neighbours.

To compute the fused matrix from *priorMatrix* and *contextMatrix*, a full and sparse kernel on the vertex set *V* is defined, which is derived from the weight matrix *W*. The full kernel is a normalized weight matrix *P*_[*n* × *n*]_ that carries the full information about the similarity of each cell to all other cells, which can be defined as:

5 $$ {P}_{ij}=\left\{\begin{array}{c}{W}_{ij}/2\sum \limits_{k\ne i}{W}_{ik},j\ne i\\ {}\kern1.75em 1/2,j=i\end{array}\right. $$

Another matrix *S*_[*n* × *n*]_ encodes the local affinity which measures the similarity of a cell to the *K* most similar cells via *K* nearest neighbours:

6 $$ {S}_{ij}=\left\{\begin{array}{c}{W}_{ij}/\sum \limits_{k\in {N}_i}{W}_{ik},j\in {N}_i\\ {}\kern3em 0,\mathrm{otherwise}\end{array}\right. $$

Here *N*_*i*_ are the neighbours of cell *c*_*i*_ including *c*_*i*_ in the graph *G*. The network fusion process always starts from *P* as the initial state using *S* as the kernel matrix to efficiently capture local structure of graphs.

To fuse the *priorMatrix* and *contextMatrix*, two similarity matrices *W*^*a*^ and *W*^*b*^ were computed, respectively. Then the initial state matrices *P*^*a*^ and *P*^*b*^ were calculated from the two similarity matrices, and the kernel matrices *S*^*a*^ and *S*^*b*^ were also computed. Given the initial two status matrices at *t* = 0, ${P}_{t=0}^a$ and ${P}_{t=0}^b$, the fusion process iteratively updates the respective similarity matrix:

$$ {P}_{t+1}^a={S}^a\times {P}_t^a\times {\left({S}^a\right)}^T $$

7 $$ {P}_{t+1}^b={S}^b\times {P}_t^b\times {\left({S}^b\right)}^T $$

Then after *t* steps, the overall status matrix can be obtained:

8 $$ {P}^o=\frac{P_t^a+{P}_t^b}{2} $$

*P*^*o*^ is the fused network of cells by comparing cells’ gene expression profiles combining both *priorMatrix* and *contextMatrix*, which can be used for downstream analyses, such as clustering, subtyping, and label prediction.

### Implementation of scNPF

To facilitate the application of our integrative framework, we have built an open-source R package called scNPF for preprocessing of scRNA-seq data. scNPF implements within a well-established framework integrating several preprocessing procedures for scRNA-seq data. The package also provides the ability to seamlessly connect different modules for more comprehensive analyses. The output of scNPF-propagation or scNPF-fusion can be directly applied on other scRNA-seq tools, such as cell type clustering tools, for downstream analyses. Users can also provide their own interaction networks for scNPF-propagation or scNPF-fusion. scNPF generates well-formatted output files to archive analysis outcomes of different modules. The scNPF package is freely available at https://github.com/BMILAB/scNPF.

### Performance evaluation

We used four performance metrics to quantify the cluster accuracy, including the ARI, Jaccard, Purity, and NMI. All these metrics are ranging from 0 to 1, with the higher value indicating the better performance. ARI is a widely-used metric for quantifying the concordance between two clustering results. ARI ranges from 0 for random clustering to 1 for perfect matching. Purity is a metric of the extent to which a cluster contains a single class. A purity score of 1 is possible by putting each data point in its own cluster. The Jaccard index is used to quantify the similarity between two data sets. An index of 1 indicates that the two data sets are identical, and an index of 0 means that there is not any common element between the two data sets. NMI is a variation of mutual information for measuring clustering accuracy, which corrects the effect of an agreement solely caused by chance. A higher NMI indicates higher clustering accuracy. ARI and Jaccard are calculated using the *adjustedRand* function in the R package clues [60]; NMI is obtained by the *compare* function in the R package igraph (https://igraph.org/r/).

Three additional internal validation metrics were also adopted to quantitatively assess the goodness of a clustering structure, which do not require external knowledge such as class labels but employ the intrinsic information of the clustering process. The DBI [61] is based on the average of ratios between the within cluster distances and the between cluster distances over all clusters. The smaller score of DBI indicates better separation of clusters. The Connectivity [62] measures the extent of observations that are placed in the same cluster as their nearest neighbours in the data space. The Dunn index [62] reflects non-linear combinations of the compactness and separation. The smaller the score of Connectivity or DBI, or the larger the score of Dunn, the better the separation is. We adopted the R package clValid [62] to calculate validation scores for Connectivity and Dunn and used the R package clusterSim [63] to obtain the score for DBI.

Cluster analyses were carried out to evaluate the performance of imputation methods or similarity metrics. The spectral clustering was implemented by the *SpectralClustering* function in the R package SNFtool [58] with the number of clusters set as the number of cell types. The hierarchical clustering [40] is performed by the *flashClust* function in the R package flashClust (method = average) [64]. The PAM clustering [42] is implemented by the *pam* function in the cluster R package [65]. The k-means clustering is implemented by the *kmeans* function of the stats R package with the maximum number of iterations set as 1e+ 9, the number of cluster centers set as the number of cell types, and the random seed set at 1000. The Rtsne package [66] is utilized to obtain the 2D embedding based on t-SNE.

## Additional files

Additional file 1: **Figure S1.** Illustration of the raw and imputed data of two randomly selected cells from the cortex fetal-quiescent of the Darmanis data. **Figure S2.** Violin plots showing expression profiles of three marker genes (a) and numbers of expressed genes (b) in the nine cell types of the Darmains data before or after imputation. **Figure S3.** Benchmarking of scNPF-propagation on eight published scRNA-seq data sets. **Figure S4.** Evaluation of the effect of paramter *r* of scNPF-propagation on two data sets, Darmanis (A) and Baron (B). **Figure S5.** Characteristics of three priori gene-gene interaction networks. **Figure S6.** Characteristics of imputed expression matrices for the Darmanis data obtained by the context mode or the priori mode with different interaction networks. **Figure S7.** Benchmarking of scNPF-propagation on eight published scRNA-seq data sets using the context mode and the priori mode with different priori networks including String, HumanNet, and INet. **Figure S8.** Benchmark results of scNPF-fusion on the Baron data. **Figure S9.** Performance comparison of the five similarity measurements on eight published scRNA-seq data sets. **Figure S10.** Benchmarking of scNPF-fusion on eight published scRNA-seq data sets. **Figure S11.** Benchmarking of scNPF-fusion on eight published scRNA-seq data sets by applying hierarchical clustering on the similarity matrices. **Figure S12.** Benchmarking of scNPF-fusion on eight published scRNA-seq data sets by applying spectral clustering on the similarity matrices. **Figure S13.** Benchmarking of scNPF-fusion on eight published scRNA-seq data sets by applying partitioning around medoids clustering on the similarity matrices. **Figure S14.** Evaluation of the effect of parameters of scNPF-fusion on two data sets, Darmanis (A) and Baron (B). **Figure S15.** Visualization of results from scNPF-fusion with different network combinations on the Darmanis data. **Figure S16.** Performance comparison of similarities learned from scNPF-fusion with different network combinations on eight published scRNA-seq data sets. **Figure S17.** Benchmarking of scNPF-fusion with different network combinations on eight published scRNA-seq data sets. (PPTX 6626 kb)

Additional file 2: **Table S1.** Benchmark scRNA-seq data sets. (XLSX 9 kb)

## Abbreviations

ARI: Adjusted rand index
DBI: Davies-Bouldin index
GEO: Gene expression omnibus
HC: Hierarchical clustering
NMI: Normalized mutual information
PAM: Partitioning around medoids
PPI: Protein-protein interaction
QC: Quality control
RWR: Random walk with restart
scNPF: An integrative scRNA-seq preprocessing framework assisted by network propagation and network fusion
scRNA-seq: Single-cell RNA sequencing
SIMLR: Single-cell interpretation via multikernel learning
SNF: Similarity network fusion
String: Search tool for the retrieval of interacting genes/proteins
t-SNE: t-distributed stochastic neighbour embedding
