# Supplementary material for: scNPF: an integrative framework assisted by network propagation and network fusion for preprocessing of single-cell RNA-seq data
Source: BMC Genomics. 2019 May 8;20:347. doi: 10.1186/s12864-019-5747-5 (PMC6505295; doi:10.1186/s12864-019-5747-5)
Supplement: Supplementary file 1 — Figure S1. Illustration of the raw and imputed data of two randomly selected cells from the cortex fetal-quiescent of the Darmanis data. Figure S2. Violin plots showing expression profiles of three marker genes (a) and numbers of expressed genes (b) in the nine cell types of the Darmains data before or after imputation. Figure S3. Benchmarking of scNPF-propagation on eight published scRNA-seq data sets. Figure S4. Evaluation of the effect of paramter r of scNPF-propagation on two data sets, Darmanis (A) and Baron (B). Figure S5. Characteristics of three priori gene-gene interaction networks. Figure S6. Characteristics of imputed expression matrices for the Darmanis data obtained by the context mode or the priori mode with different interaction networks. Figure S7. Benchmarking of scNPF-propagation on eight published scRNA-seq data sets using the context mode and the priori mode with different priori networks including String, HumanNet, and INet. Figure S8. Benchmark results of scNPF-fusion on the Baron data. Figure S9. Performance comparison of the five similarity measurements on eight published scRNA-seq data sets. Figure S10. Benchmarking of scNPF-fusion on eight published scRNA-seq data sets. Figure S11. Benchmarking of scNPF-fusion on eight published scRNA-seq data sets by applying hierarchical clustering on the similarity matrices. Figure S12. Benchmarking of scNPF-fusion on eight published scRNA-seq data sets by applying spectral clustering on the similarity matrices. Figure S13. Benchmarking of scNPF-fusion on eight published scRNA-seq data sets by applying partitioning around medoids clustering on the similarity matrices. Figure S14. Evaluation of the effect of parameters of scNPF-fusion on two data sets, Darmanis (A) and Baron (B). Figure S15. Visualization of results from scNPF-fusion with different network combinations on the Darmanis data. Figure S16. Performance comparison of similarities learned from scNPF-fusion with different network combinations on [file 12864_2019_5747_MOESM1_ESM.pptx]

## Slide 1
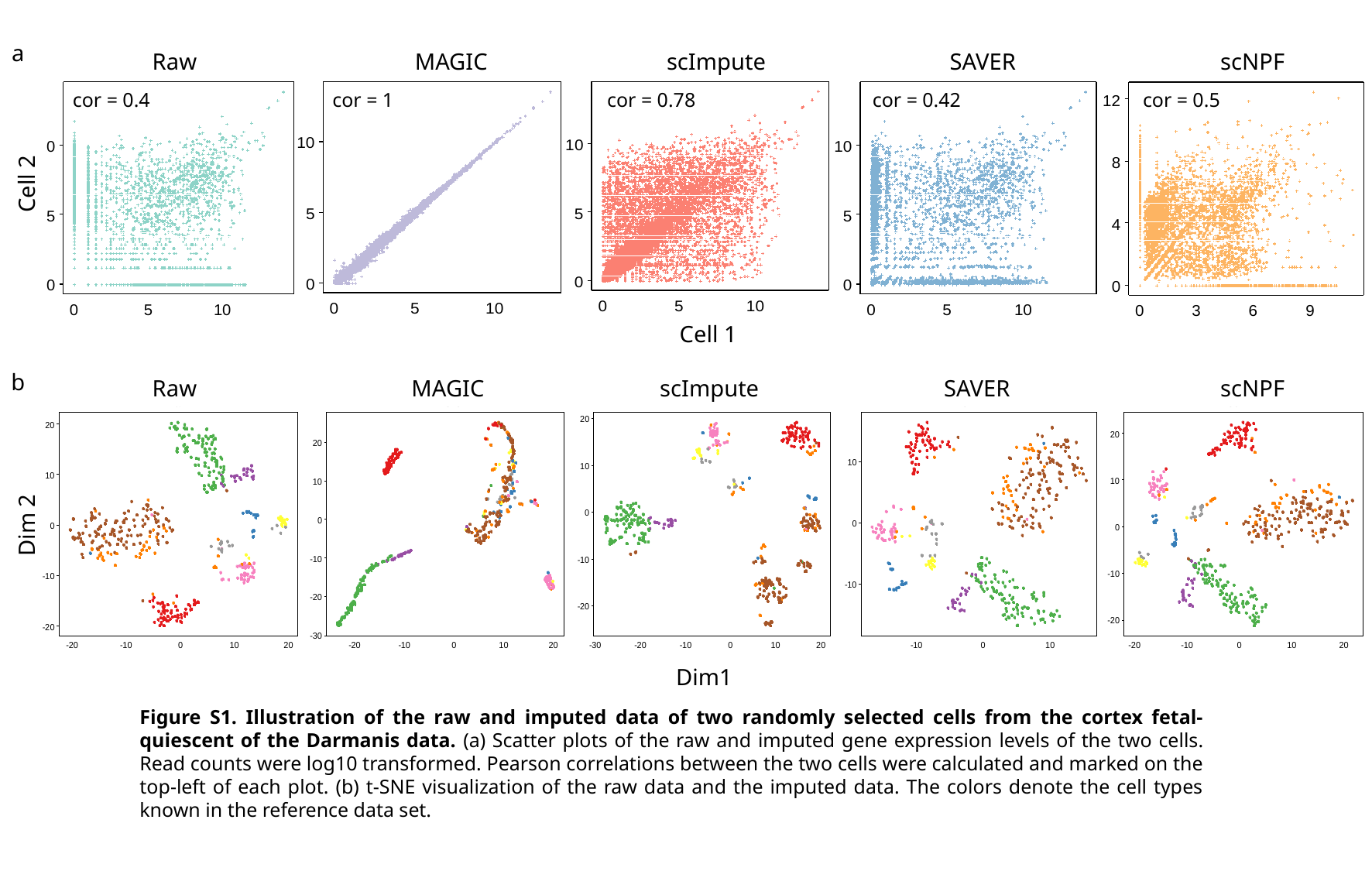

a
Raw
MAGIC
scImpute
SAVER
scNPF
cor = 0.4
cor = 1
cor = 0.78
cor = 0.42
cor = 0.5
Cell 2
Cell 1
b
Raw
MAGIC
scImpute
SAVER
scNPF
Dim 2
Dim1
Figure S1. Illustration of the raw and imputed data of two randomly selected cells from the cortex fetal-quiescent of the Darmanis data. (a) Scatter plots of the raw and imputed gene expression levels of the two cells. Read counts were log10 transformed. Pearson correlations between the two cells were calculated and marked on the top-left of each plot. (b) t-SNE visualization of the raw data and the imputed data. The colors denote the cell types known in the reference data set.

## Slide 2
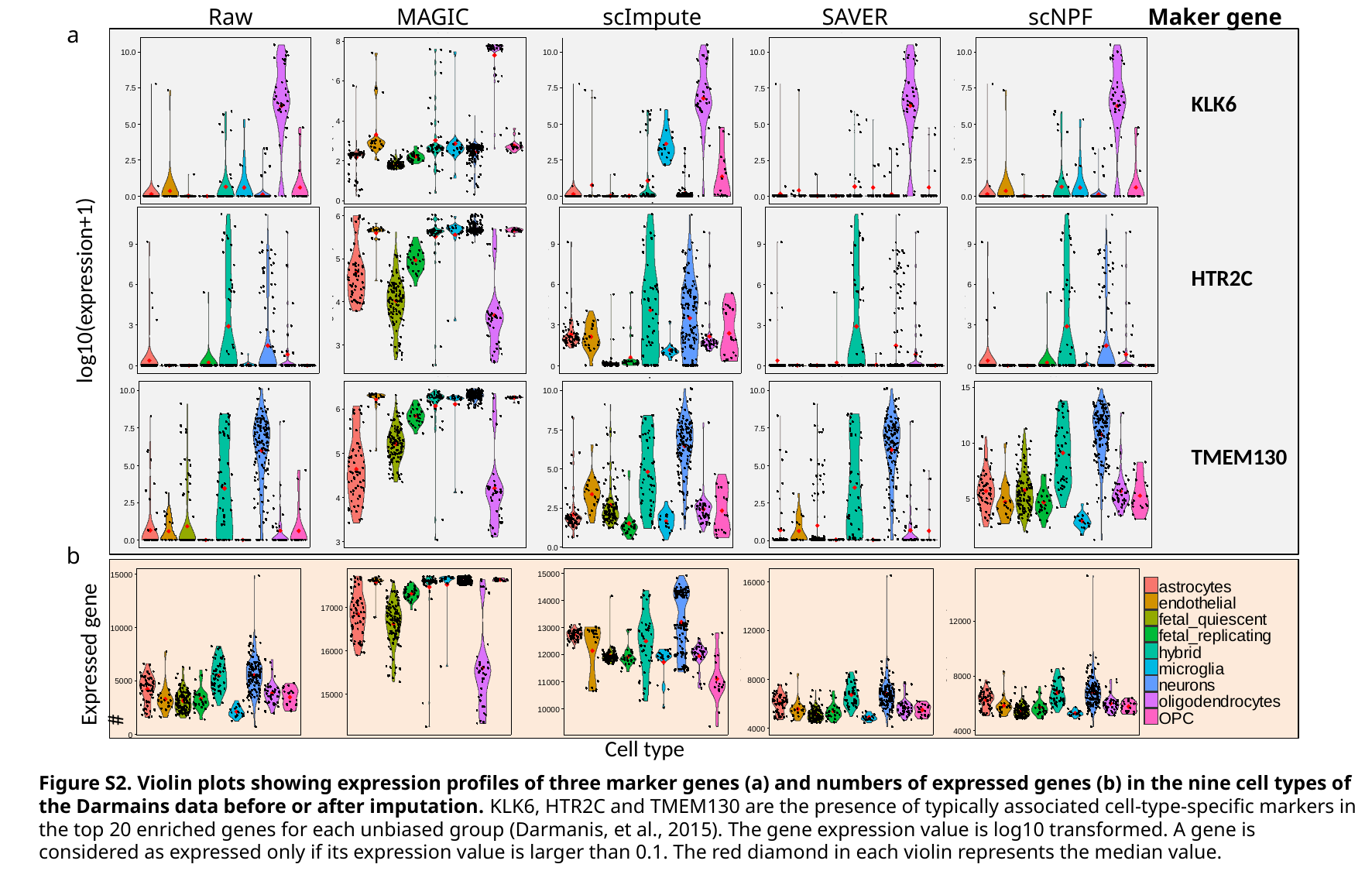

Raw
MAGIC
scImpute
SAVER
scNPF
Maker gene
a
KLK6
log10(expression+1)
HTR2C
TMEM130
b
Expressed gene #
Cell type
Figure S2. Violin plots showing expression profiles of three marker genes (a) and numbers of expressed genes (b) in the nine cell types of the Darmains data before or after imputation. KLK6, HTR2C and TMEM130 are the presence of typically associated cell-type-specific markers in the top 20 enriched genes for each unbiased group (Darmanis, et al., 2015). The gene expression value is log10 transformed. A gene is considered as expressed only if its expression value is larger than 0.1. The red diamond in each violin represents the median value.

## Slide 3
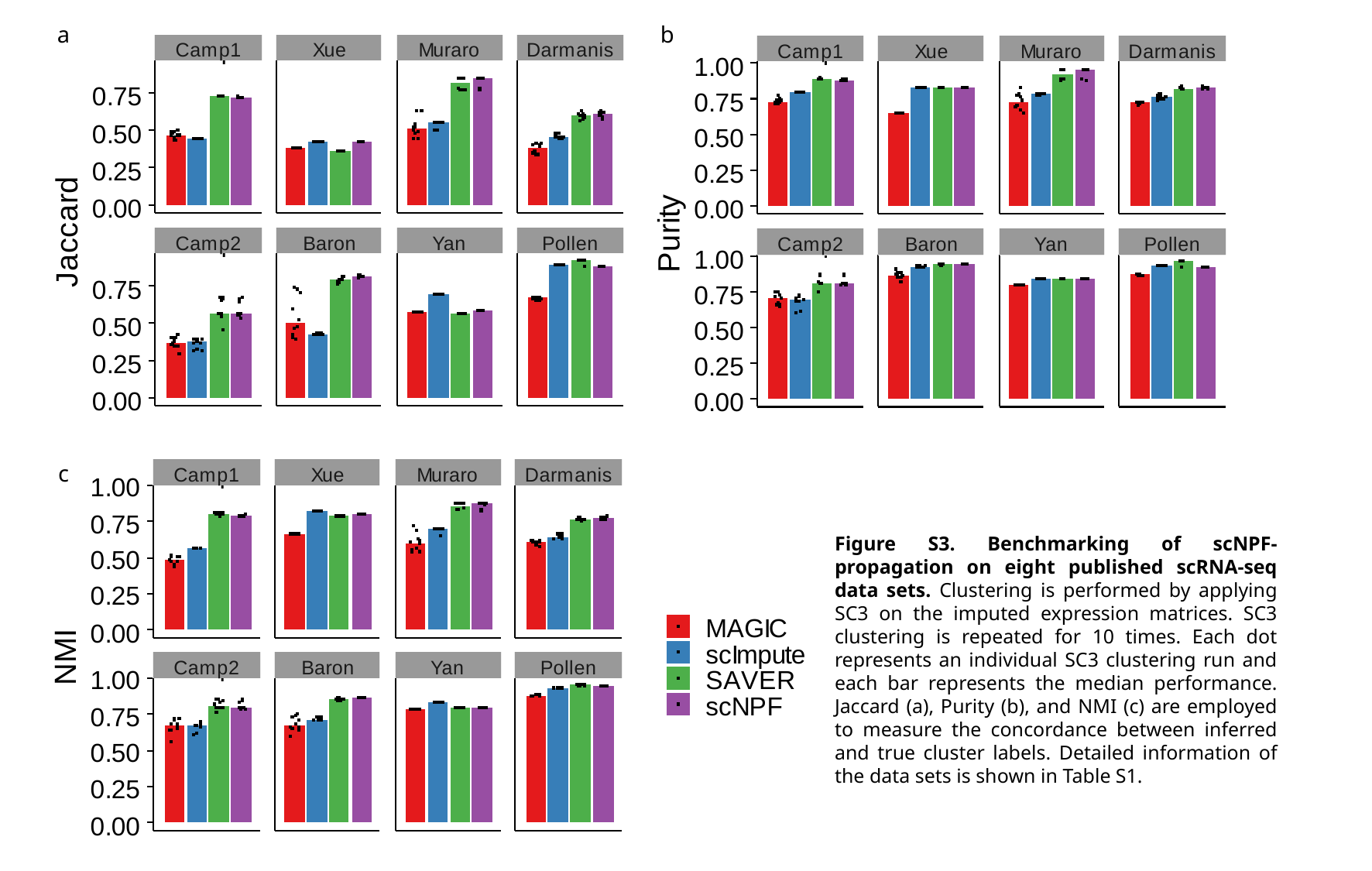

a
b
c
Figure S3. Benchmarking of scNPF-propagation on eight published scRNA-seq data sets. Clustering is performed by applying SC3 on the imputed expression matrices. SC3 clustering is repeated for 10 times. Each dot represents an individual SC3 clustering run and each bar represents the median performance. Jaccard (a), Purity (b), and NMI (c) are employed to measure the concordance between inferred and true cluster labels. Detailed information of the data sets is shown in Table S1.

## Slide 4
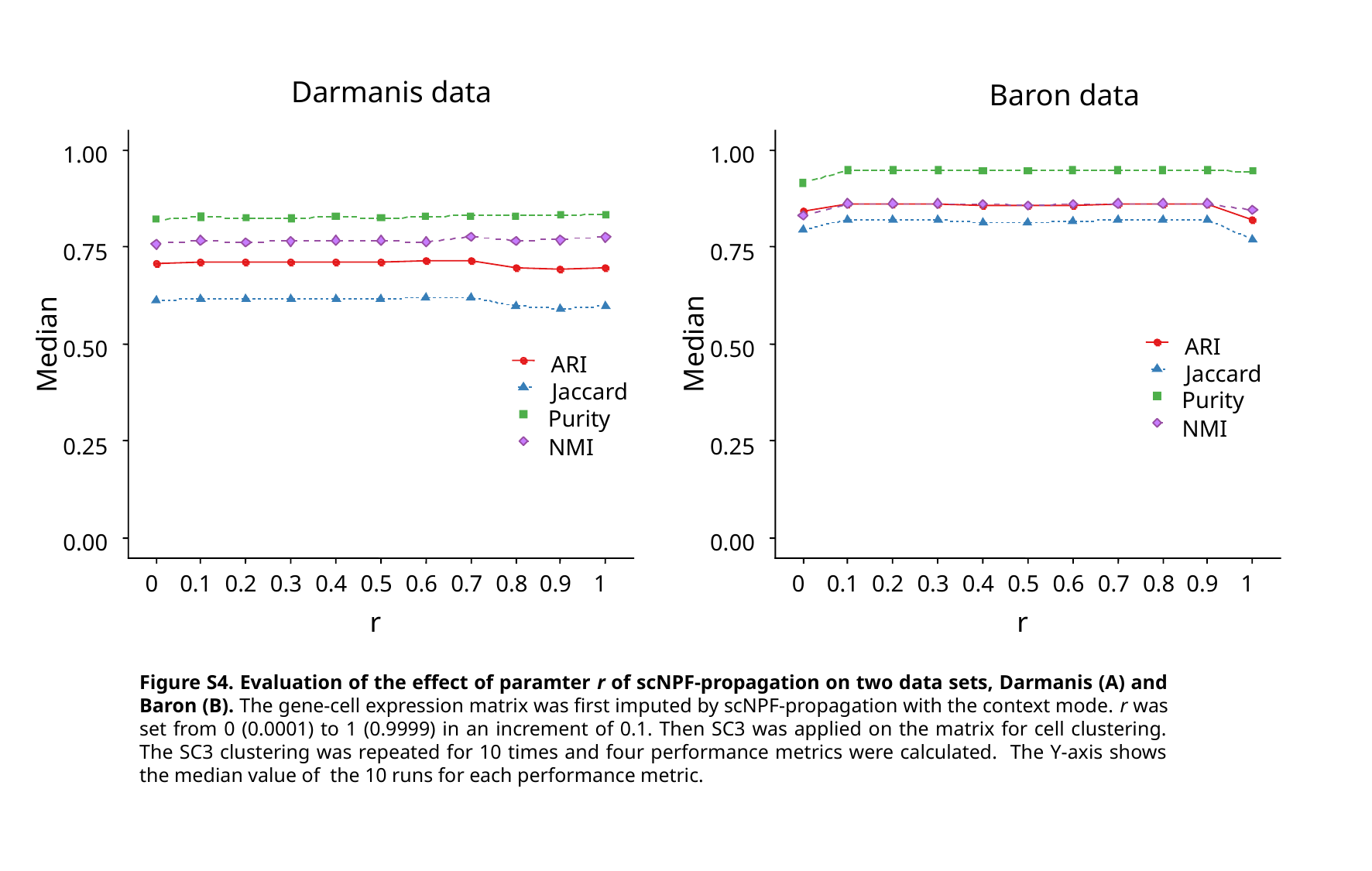

Darmanis data
Baron data
1.00
1.00
0.75
0.75
Median
Median
ARI
Jaccard
Purity
NMI
0.50
0.50
ARI
Jaccard
Purity
NMI
0.25
0.25
0.00
0.00
0
0.1
0.2
0.3
0.4
0.5
0.6
0.7
0.8
0.9
1
0
0.1
0.2
0.3
0.4
0.5
0.6
0.7
0.8
0.9
1
r
r
Figure S4. Evaluation of the effect of paramter r of scNPF-propagation on two data sets, Darmanis (A) and Baron (B). The gene-cell expression matrix was first imputed by scNPF-propagation with the context mode. r was set from 0 (0.0001) to 1 (0.9999) in an increment of 0.1. Then SC3 was applied on the matrix for cell clustering. The SC3 clustering was repeated for 10 times and four performance metrics were calculated. The Y-axis shows the median value of the 10 runs for each performance metric.

## Slide 5
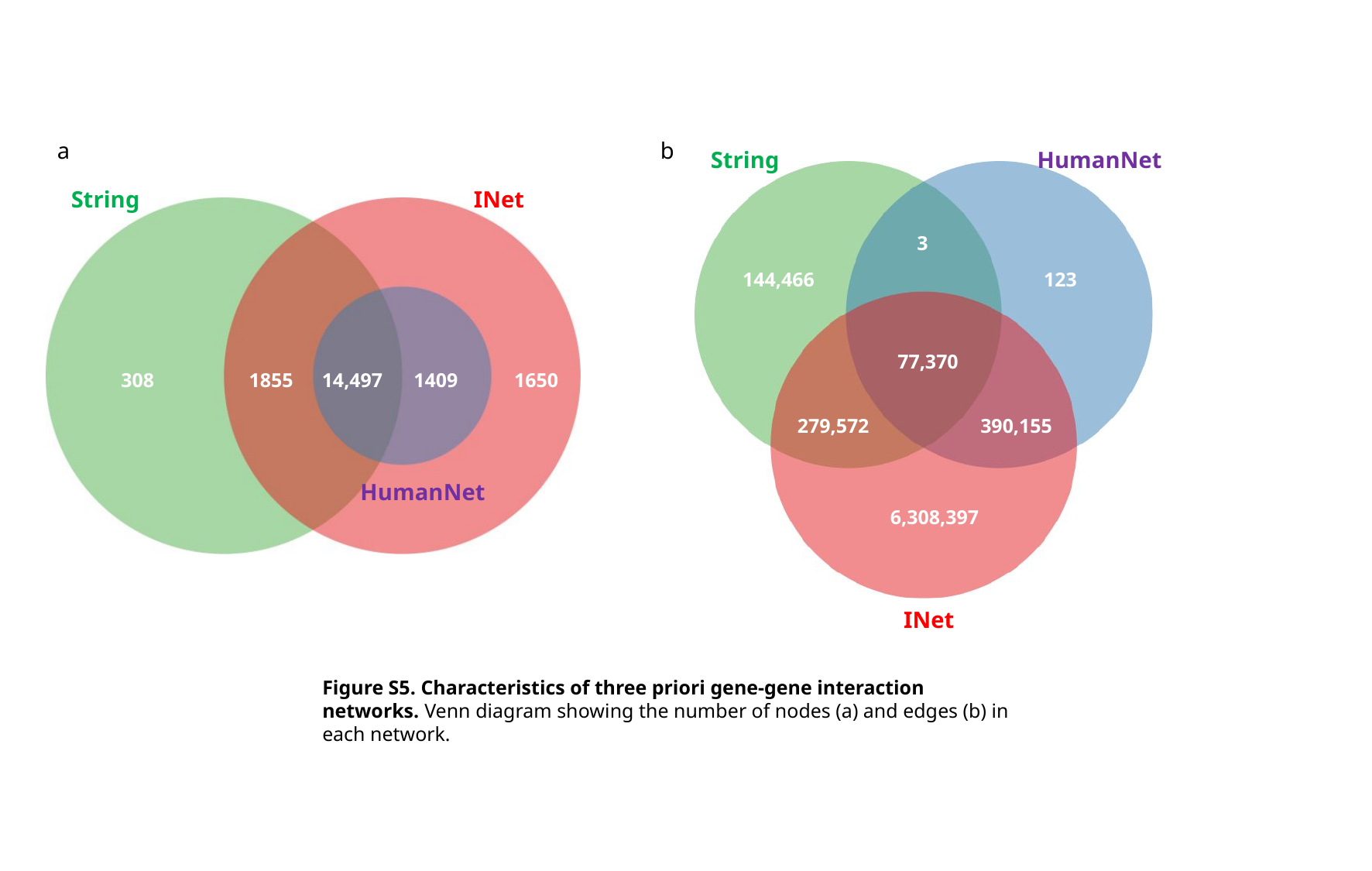

a
b
String
HumanNet
String
INet
3
144,466
123
77,370
308
1855
14,497
1409
1650
279,572
390,155
HumanNet
6,308,397
INet
Figure S5. Characteristics of three priori gene-gene interaction networks. Venn diagram showing the number of nodes (a) and edges (b) in each network.

## Slide 6
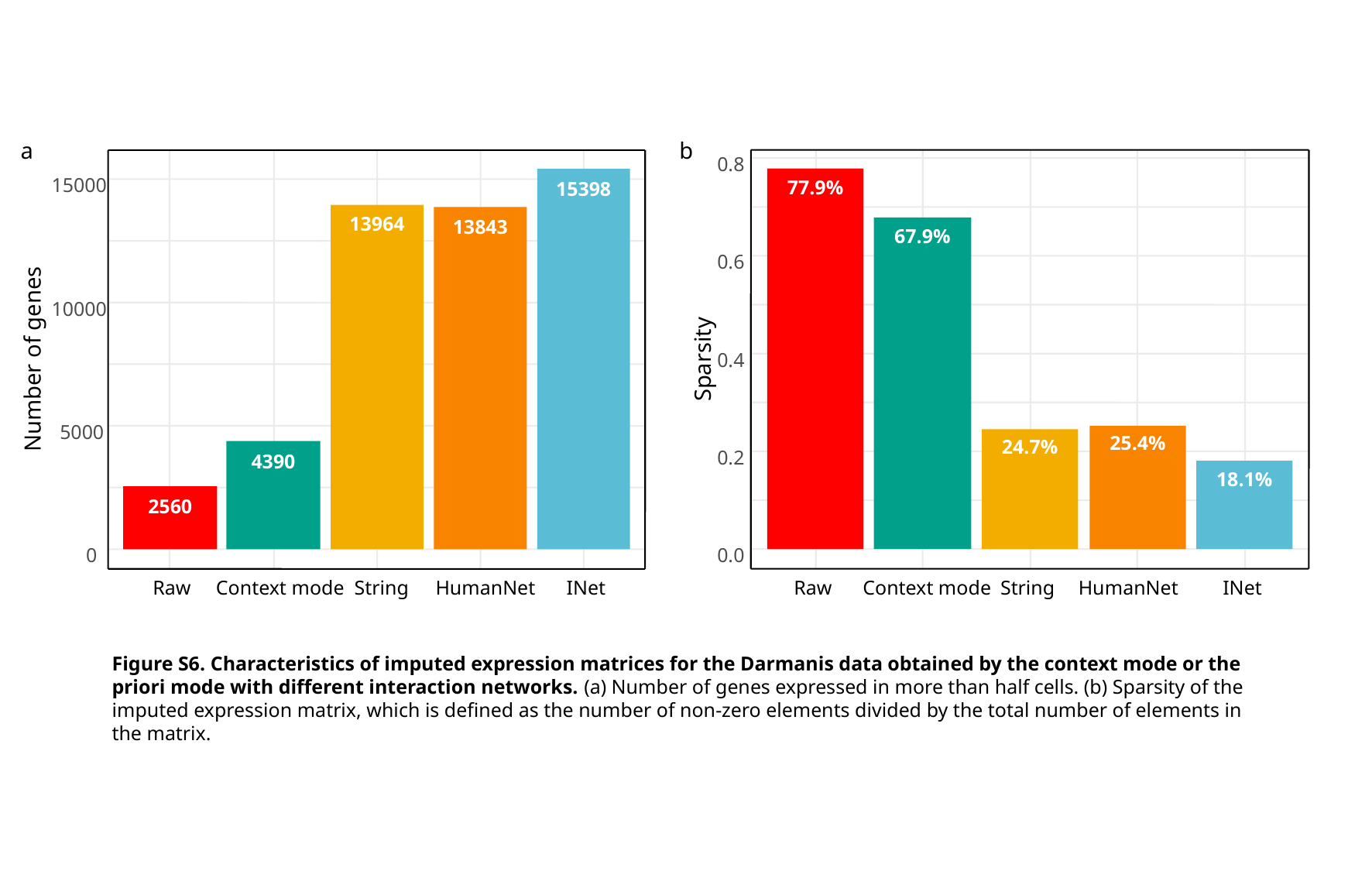

a
b
0.8
77.9%
67.9%
0.6
Sparsity
0.4
25.4%
24.7%
0.2
18.1%
0.0
Raw
Context mode
String
HumanNet
INet
15000
15398
13964
13843
10000
Number of genes
5000
4390
2560
0
Raw
Context mode
String
HumanNet
INet
Figure S6. Characteristics of imputed expression matrices for the Darmanis data obtained by the context mode or the priori mode with different interaction networks. (a) Number of genes expressed in more than half cells. (b) Sparsity of the imputed expression matrix, which is defined as the number of non-zero elements divided by the total number of elements in the matrix.

## Slide 7
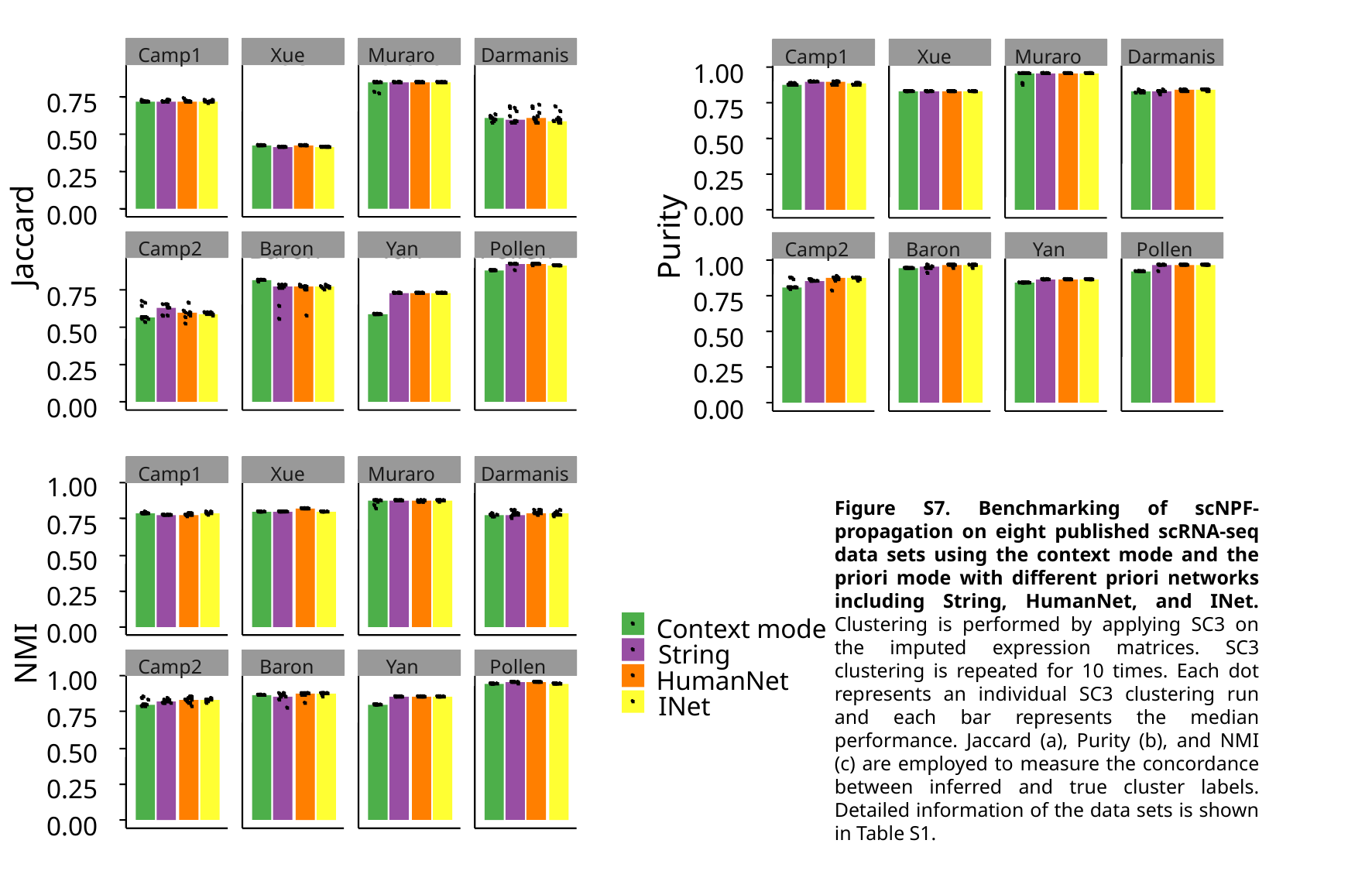

1.00
0.75
0.50
0.25
0.00
Purity
1.00
0.75
0.50
0.25
0.00
Camp2
Baron
Yan
Camp1
Xue
Muraro
Darmanis
Pollen
Xue
Muraro
0.75
0.50
0.25
0.00
Jaccard
Baron
Yan
Pollen
0.75
0.50
0.25
0.00
Camp1
Xue
Muraro
Darmanis
Camp2
Baron
Yan
Pollen
1.00
0.75
0.50
0.25
Context mode
0.00
NMI
String
1.00
HumanNet
INet
0.75
0.50
0.25
0.00
Camp1
Xue
Muraro
Darmanis
Camp2
Baron
Yan
Pollen
Figure S7. Benchmarking of scNPF-propagation on eight published scRNA-seq data sets using the context mode and the priori mode with different priori networks including String, HumanNet, and INet. Clustering is performed by applying SC3 on the imputed expression matrices. SC3 clustering is repeated for 10 times. Each dot represents an individual SC3 clustering run and each bar represents the median performance. Jaccard (a), Purity (b), and NMI (c) are employed to measure the concordance between inferred and true cluster labels. Detailed information of the data sets is shown in Table S1.

## Slide 8
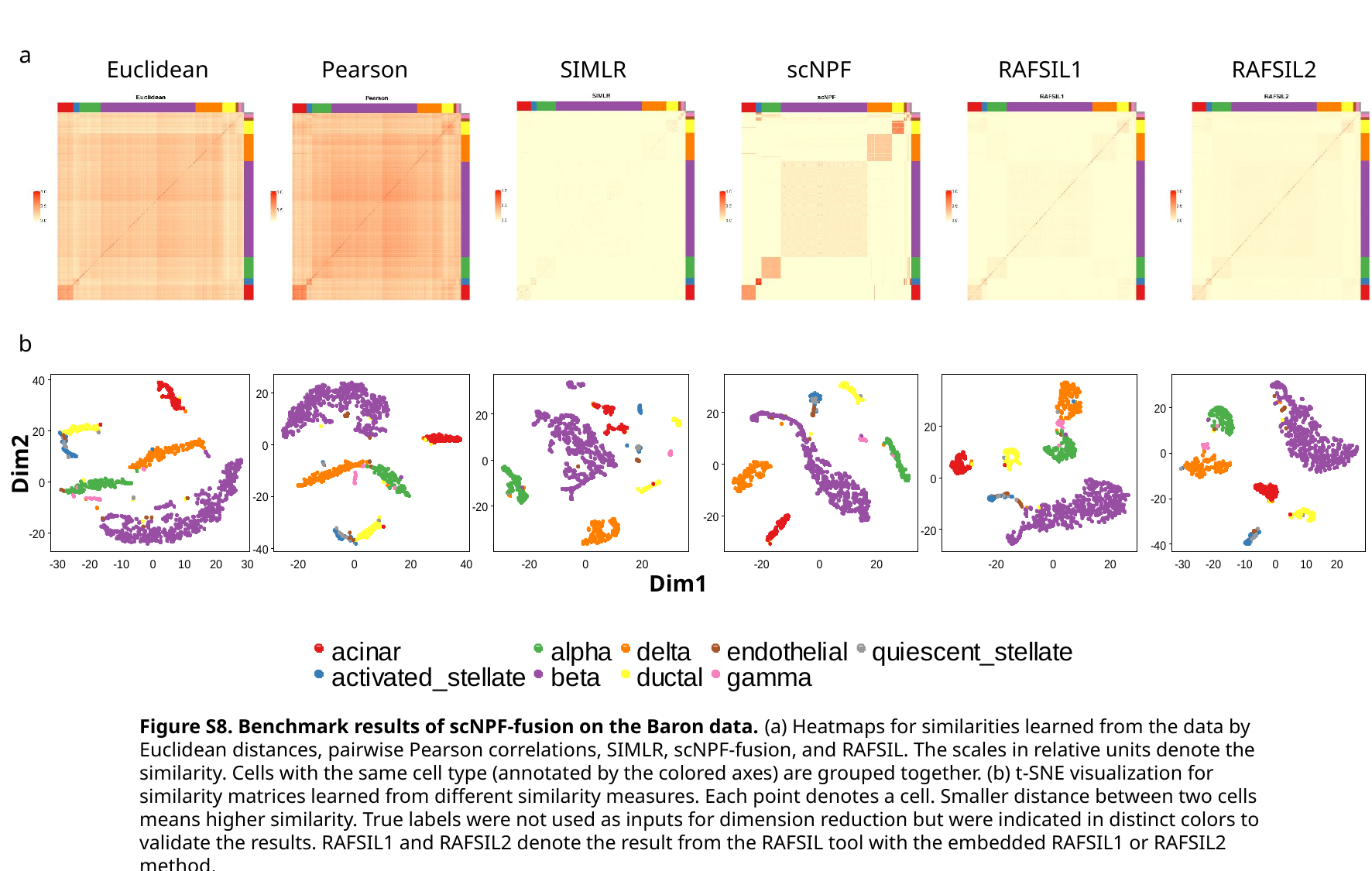

a
Euclidean
Pearson
SIMLR
scNPF
RAFSIL1
RAFSIL2
b
Dim2
Dim1
Figure S8. Benchmark results of scNPF-fusion on the Baron data. (a) Heatmaps for similarities learned from the data by Euclidean distances, pairwise Pearson correlations, SIMLR, scNPF-fusion, and RAFSIL. The scales in relative units denote the similarity. Cells with the same cell type (annotated by the colored axes) are grouped together. (b) t-SNE visualization for similarity matrices learned from different similarity measures. Each point denotes a cell. Smaller distance between two cells means higher similarity. True labels were not used as inputs for dimension reduction but were indicated in distinct colors to validate the results. RAFSIL1 and RAFSIL2 denote the result from the RAFSIL tool with the embedded RAFSIL1 or RAFSIL2 method.

## Slide 9
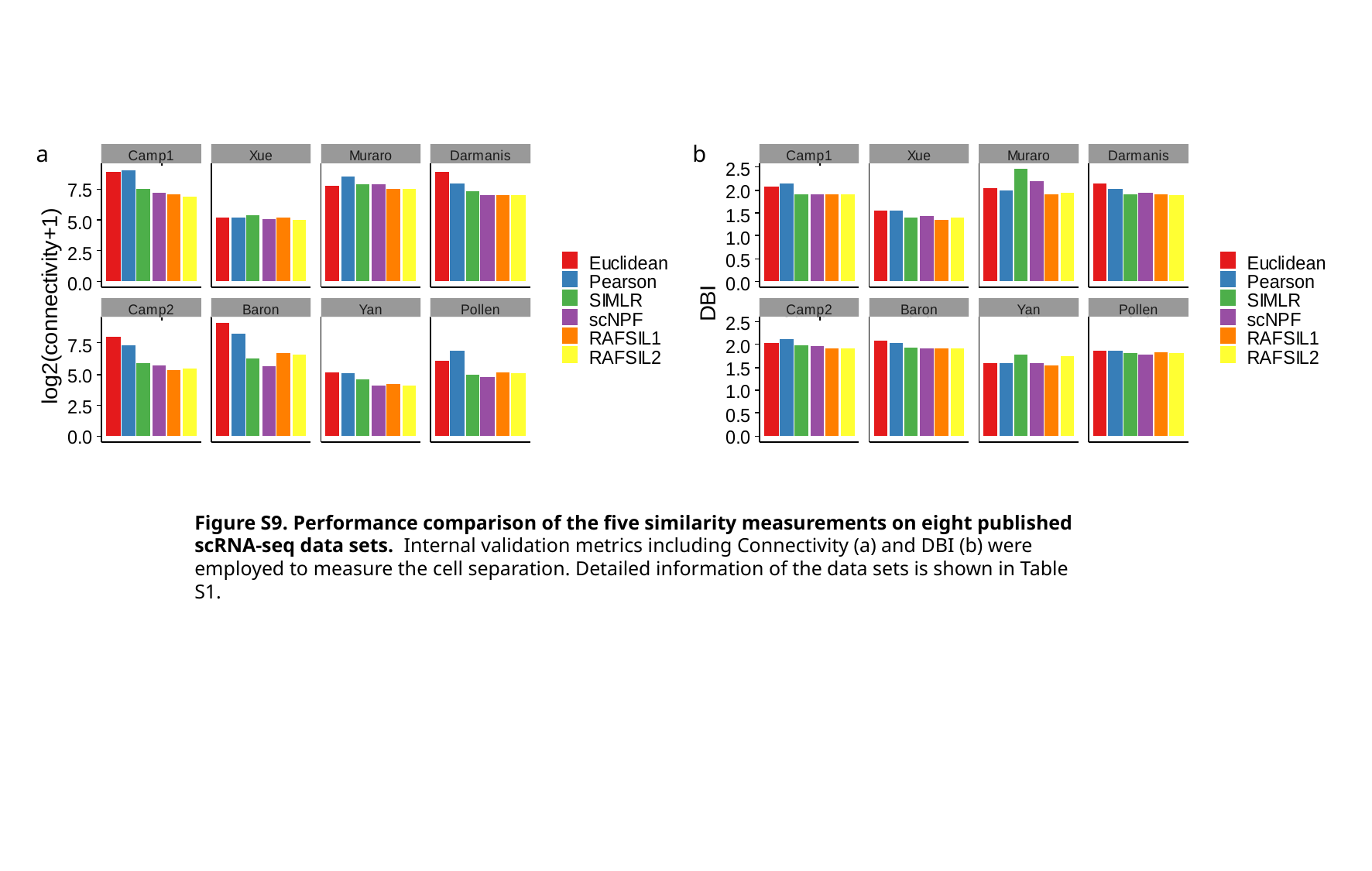

a
b
Figure S9. Performance comparison of the five similarity measurements on eight published scRNA-seq data sets. Internal validation metrics including Connectivity (a) and DBI (b) were employed to measure the cell separation. Detailed information of the data sets is shown in Table S1.

## Slide 10
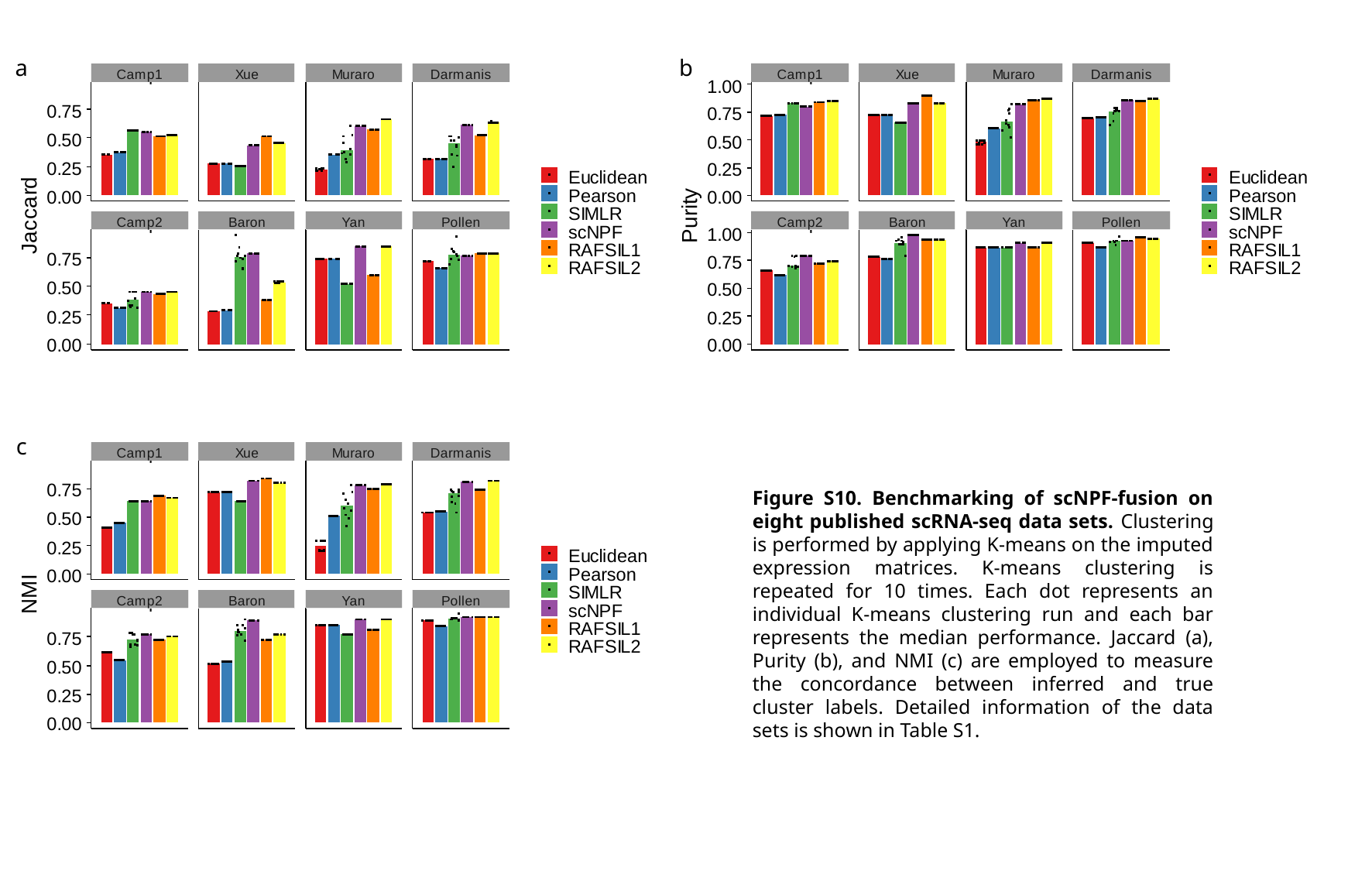

a
b
c
Figure S10. Benchmarking of scNPF-fusion on eight published scRNA-seq data sets. Clustering is performed by applying K-means on the imputed expression matrices. K-means clustering is repeated for 10 times. Each dot represents an individual K-means clustering run and each bar represents the median performance. Jaccard (a), Purity (b), and NMI (c) are employed to measure the concordance between inferred and true cluster labels. Detailed information of the data sets is shown in Table S1.

## Slide 11
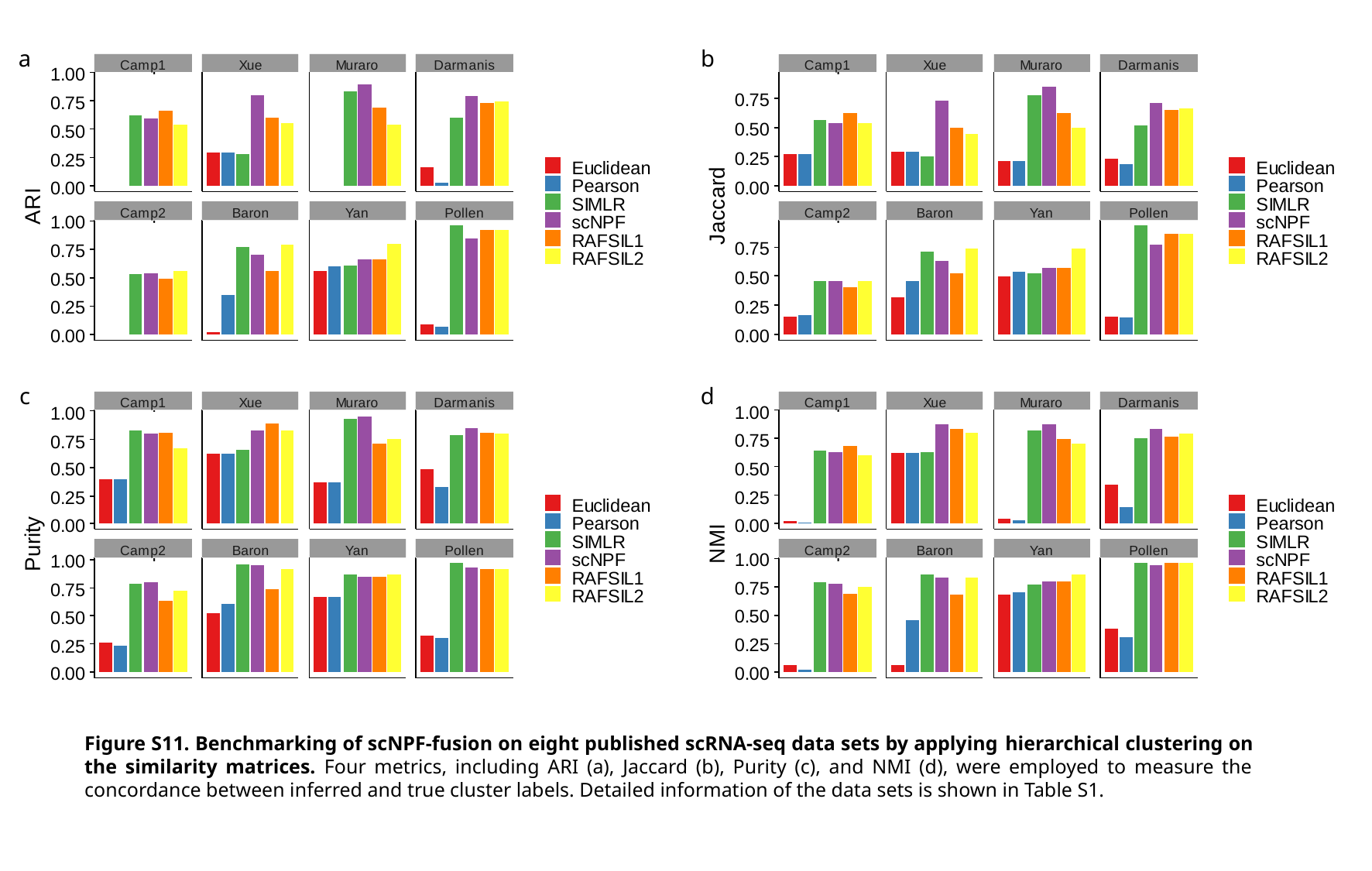

a
b
c
d
Figure S11. Benchmarking of scNPF-fusion on eight published scRNA-seq data sets by applying hierarchical clustering on the similarity matrices. Four metrics, including ARI (a), Jaccard (b), Purity (c), and NMI (d), were employed to measure the concordance between inferred and true cluster labels. Detailed information of the data sets is shown in Table S1.

## Slide 12
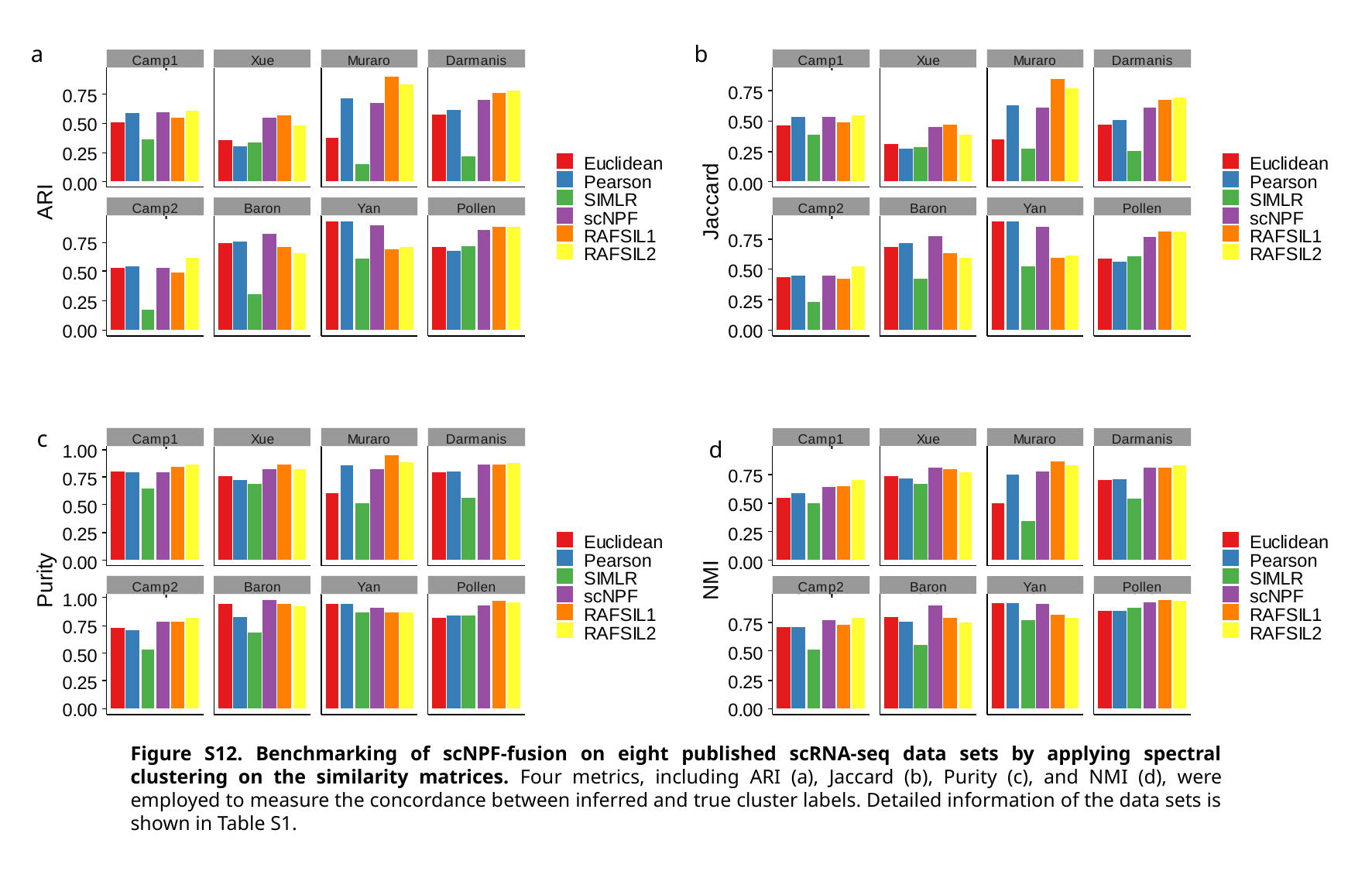

a
b
c
d
Figure S12. Benchmarking of scNPF-fusion on eight published scRNA-seq data sets by applying spectral clustering on the similarity matrices. Four metrics, including ARI (a), Jaccard (b), Purity (c), and NMI (d), were employed to measure the concordance between inferred and true cluster labels. Detailed information of the data sets is shown in Table S1.

## Slide 13
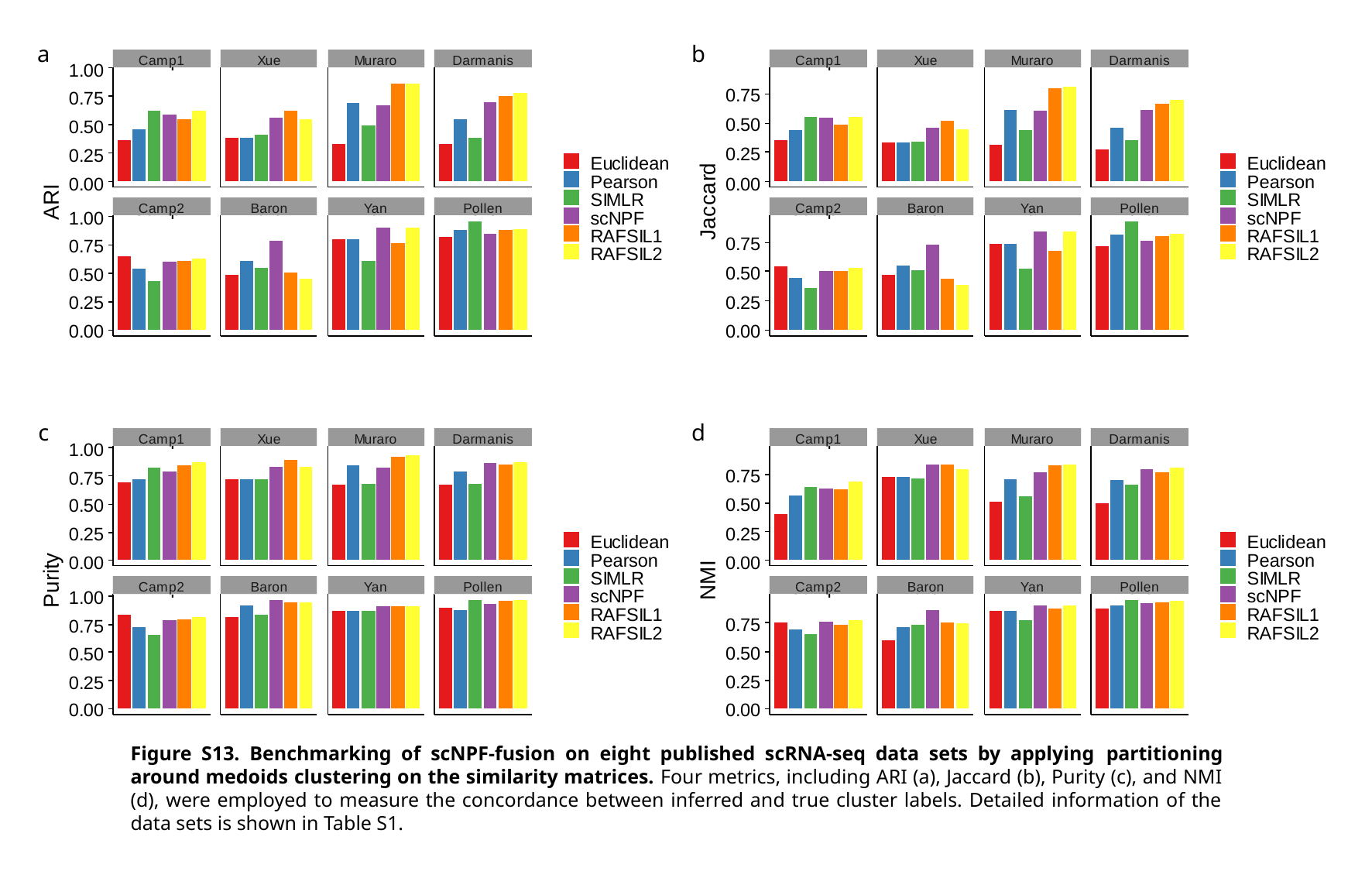

a
b
c
d
Figure S13. Benchmarking of scNPF-fusion on eight published scRNA-seq data sets by applying partitioning around medoids clustering on the similarity matrices. Four metrics, including ARI (a), Jaccard (b), Purity (c), and NMI (d), were employed to measure the concordance between inferred and true cluster labels. Detailed information of the data sets is shown in Table S1.

## Slide 14
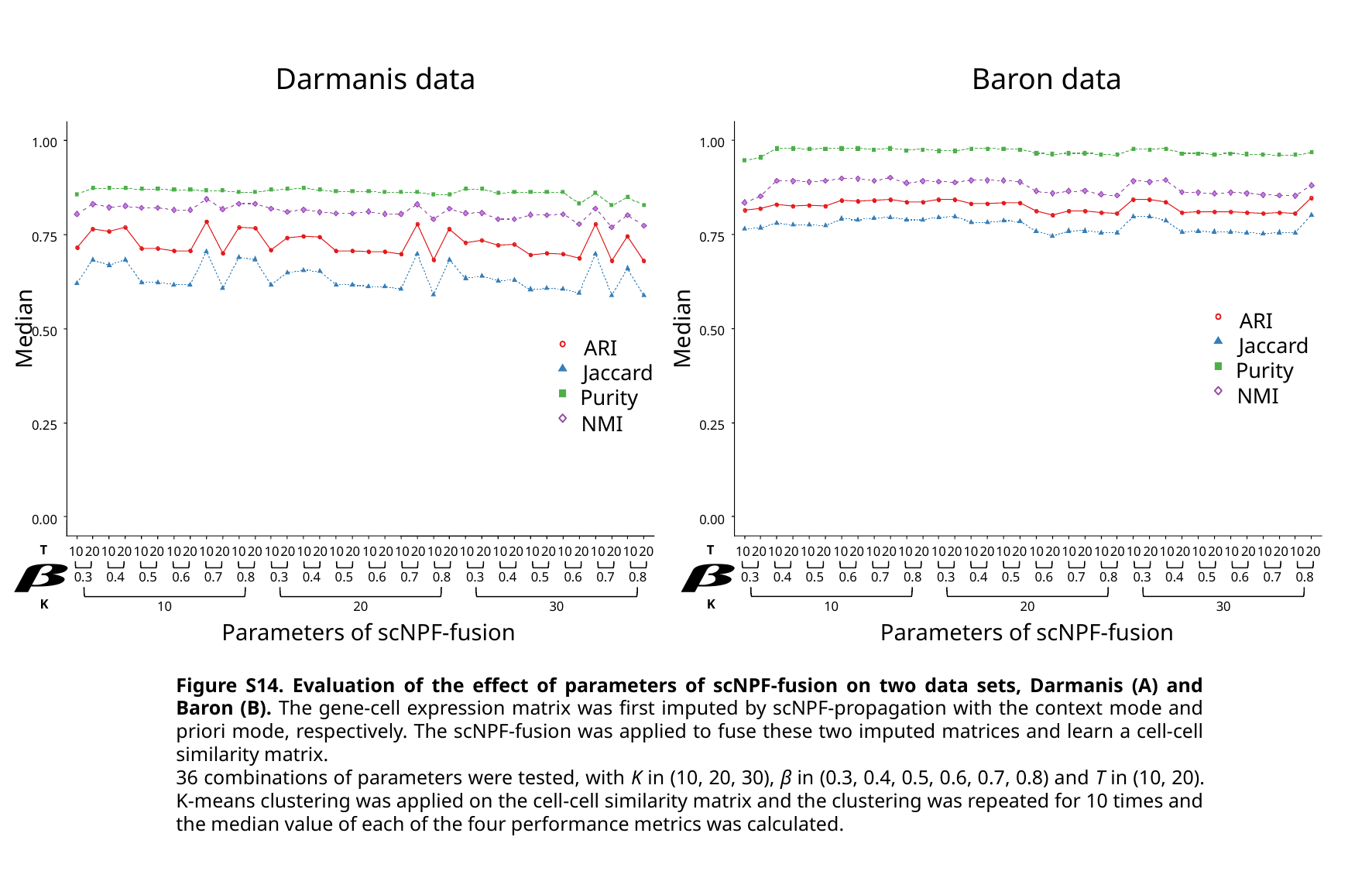

Darmanis data
Baron data
1.00
0.75
Median
0.50
0.25
0.00
Parameters of scNPF-fusion
T
10
20
10
20
10
20
10
20
10
20
10
20
10
20
10
20
10
20
10
20
10
20
10
20
10
20
10
20
10
20
10
20
10
20
10
20
0.3
0.4
0.5
0.6
0.7
0.8
10
0.3
0.4
0.5
0.6
0.7
0.8
20
0.3
0.4
0.5
0.6
0.7
0.8
30
K
1.00
0.75
ARI
Jaccard
Purity
NMI
Median
0.50
ARI
Jaccard
Purity
NMI
0.25
0.00
T
10
20
10
20
10
20
10
20
10
20
10
20
10
20
10
20
10
20
10
20
10
20
10
20
10
20
10
20
10
20
10
20
10
20
10
20
0.3
0.4
0.5
0.6
0.7
0.8
10
0.3
0.4
0.5
0.6
0.7
0.8
20
0.3
0.4
0.5
0.6
0.7
0.8
30
K
Parameters of scNPF-fusion
Figure S14. Evaluation of the effect of parameters of scNPF-fusion on two data sets, Darmanis (A) and Baron (B). The gene-cell expression matrix was first imputed by scNPF-propagation with the context mode and priori mode, respectively. The scNPF-fusion was applied to fuse these two imputed matrices and learn a cell-cell similarity matrix.
36 combinations of parameters were tested, with K in (10, 20, 30), β in (0.3, 0.4, 0.5, 0.6, 0.7, 0.8) and T in (10, 20). K-means clustering was applied on the cell-cell similarity matrix and the clustering was repeated for 10 times and the median value of each of the four performance metrics was calculated.

## Slide 15
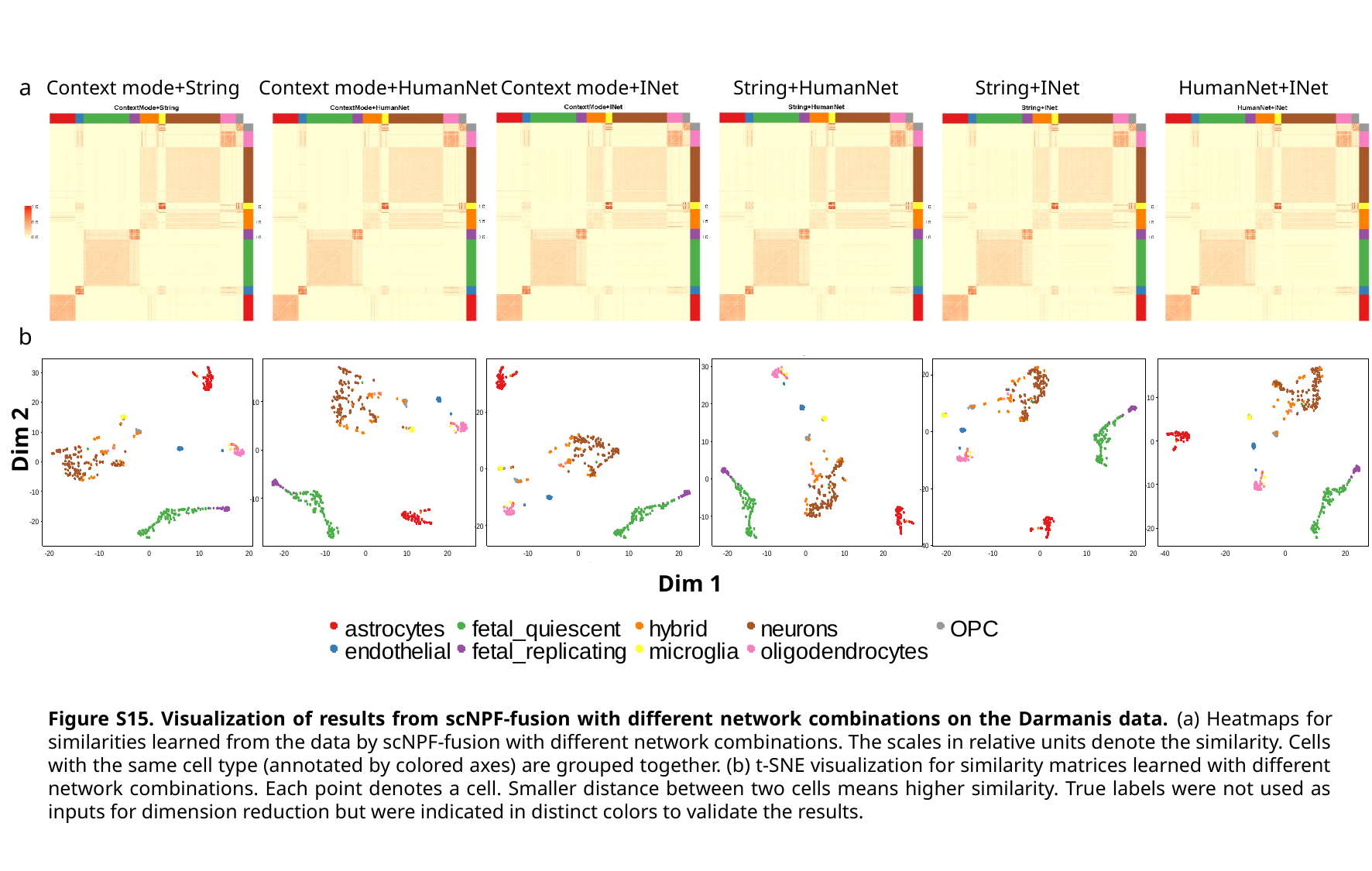

a
Context mode+String
Context mode+HumanNet
Context mode+INet
String+HumanNet
String+INet
HumanNet+INet
b
Dim 2
Dim 1
Figure S15. Visualization of results from scNPF-fusion with different network combinations on the Darmanis data. (a) Heatmaps for similarities learned from the data by scNPF-fusion with different network combinations. The scales in relative units denote the similarity. Cells with the same cell type (annotated by colored axes) are grouped together. (b) t-SNE visualization for similarity matrices learned with different network combinations. Each point denotes a cell. Smaller distance between two cells means higher similarity. True labels were not used as inputs for dimension reduction but were indicated in distinct colors to validate the results.

## Slide 16
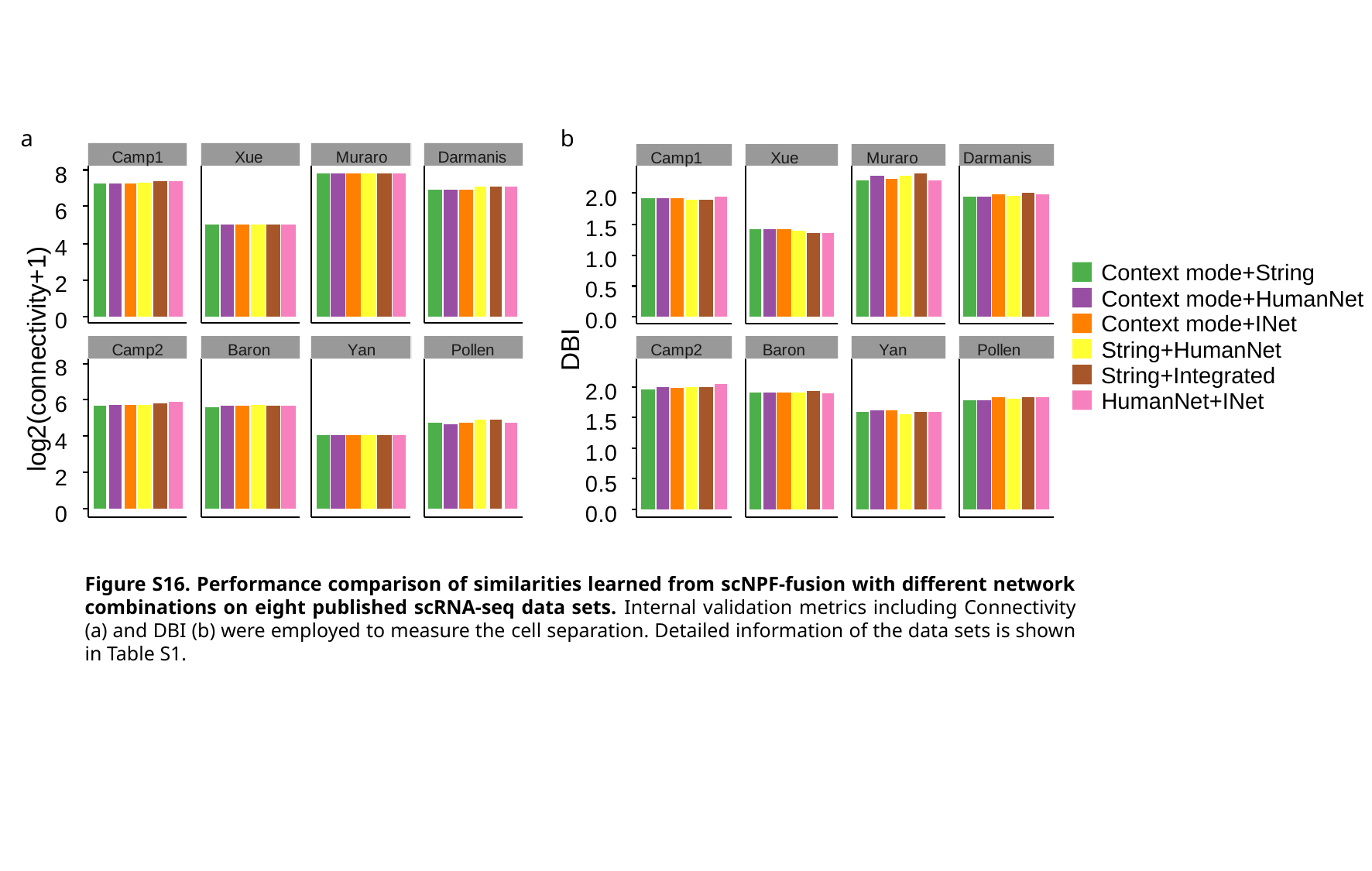

a
b
Context mode+String
Context mode+HumanNet
Context mode+INet
String+HumanNet
String+Integrated
HumanNet+INet
Figure S16. Performance comparison of similarities learned from scNPF-fusion with different network combinations on eight published scRNA-seq data sets. Internal validation metrics including Connectivity (a) and DBI (b) were employed to measure the cell separation. Detailed information of the data sets is shown in Table S1.

## Slide 17
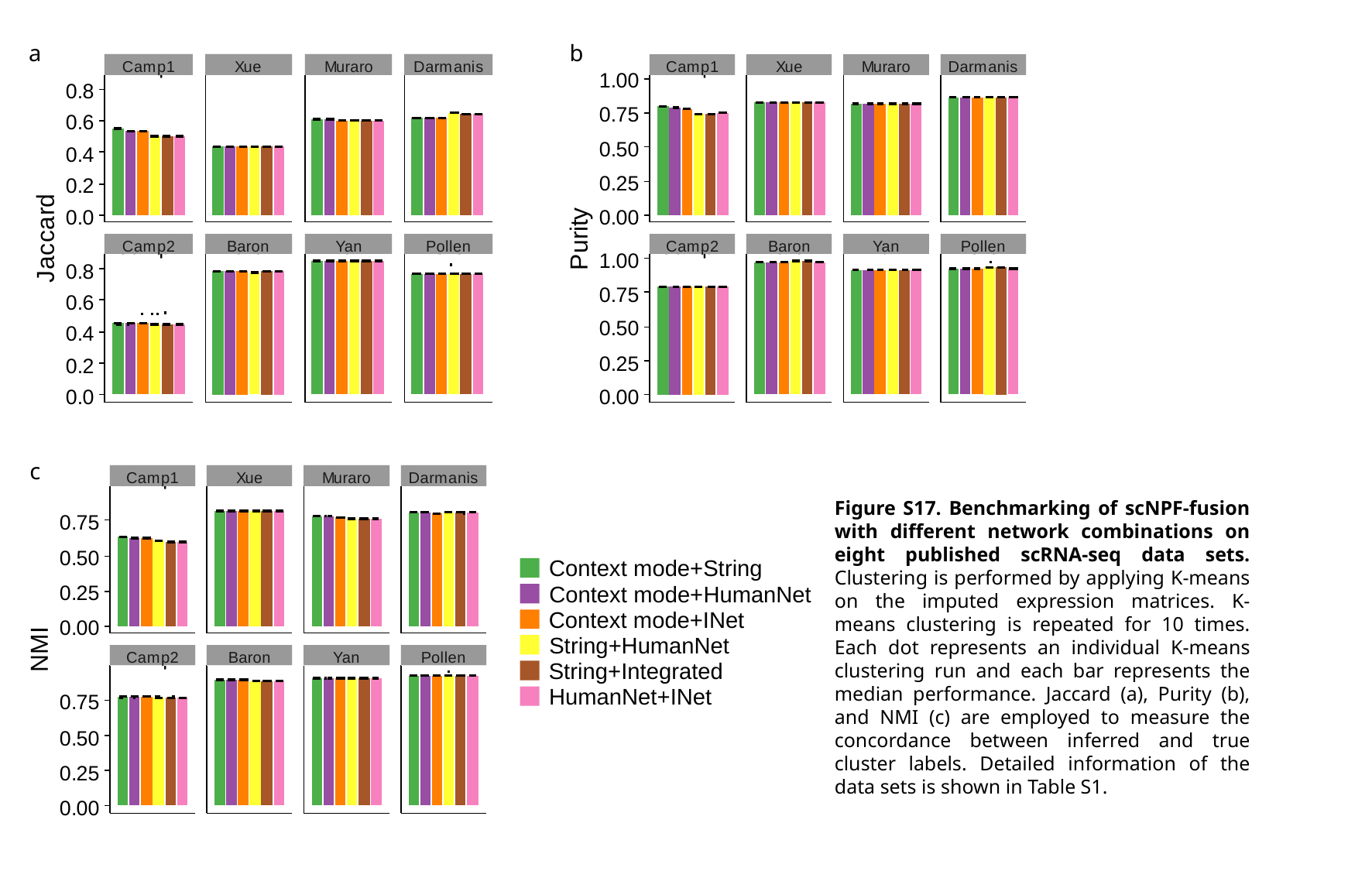

a
b
c
Figure S17. Benchmarking of scNPF-fusion with different network combinations on eight published scRNA-seq data sets. Clustering is performed by applying K-means on the imputed expression matrices. K-means clustering is repeated for 10 times. Each dot represents an individual K-means clustering run and each bar represents the median performance. Jaccard (a), Purity (b), and NMI (c) are employed to measure the concordance between inferred and true cluster labels. Detailed information of the data sets is shown in Table S1.
Context mode+String
Context mode+HumanNet
Context mode+INet
String+HumanNet
String+Integrated
HumanNet+INet
